# Beauty in Chemistry: Making Artistic Molecules with Schiff Bases

**DOI:** 10.1021/acs.joc.0c01420

**Published:** 2020-08-31

**Authors:** Luigi Fabbrizzi

**Affiliations:** Dipartimento di Chimica, Università di Pavia, 27100 Pavia, Italy

## Abstract

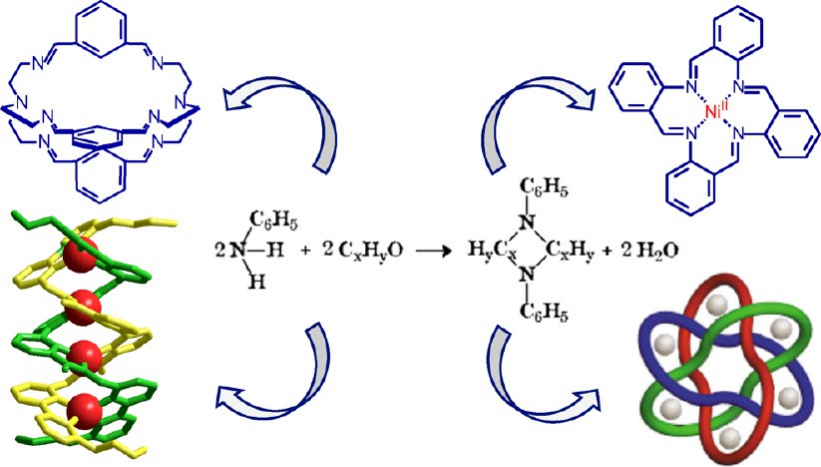

In 1864, Hugo Schiff, aged 30, discovered the reaction of aromatic aldehydes with primary amines to give imine derivatives. A C=N imine bond presents the unique properties of being strong, as expected for a covalent double bond, and of being reversible due to a fast hydrolytic process. In view of such features, Schiff base condensations are thermodynamically controlled, which, in the case of reactions involving multifunctional aldehydes and primary amines, allow the formation of complex and sophisticated structures through a trial-and-error mechanism. Back hydrolysis can be prevented by hydrogenating C=N bonds under mild conditions. In such a way, stable rings and cages of varying sizes can be synthesized. Moreover, transition and post-transition metal ions, establishing coordinative interactions with imine nitrogen atoms, can address Schiff base condensations of even more complex molecular systems, whose structure is controlled by the geometrical preferences of the metal. Metal template Schiff base condensations have produced multinuclear metal complexes exhibiting the shape of tetrahedral containers, of double helices, and, supreme wonder, of the Borromean rings. These molecular objects cannot be compared to the masterpieces of painting and sculpture of the macroscopic world, but they instill in the viewer aesthetical pleasure and admiration for their creators.

## 1 The Origins

**Figure 1:**
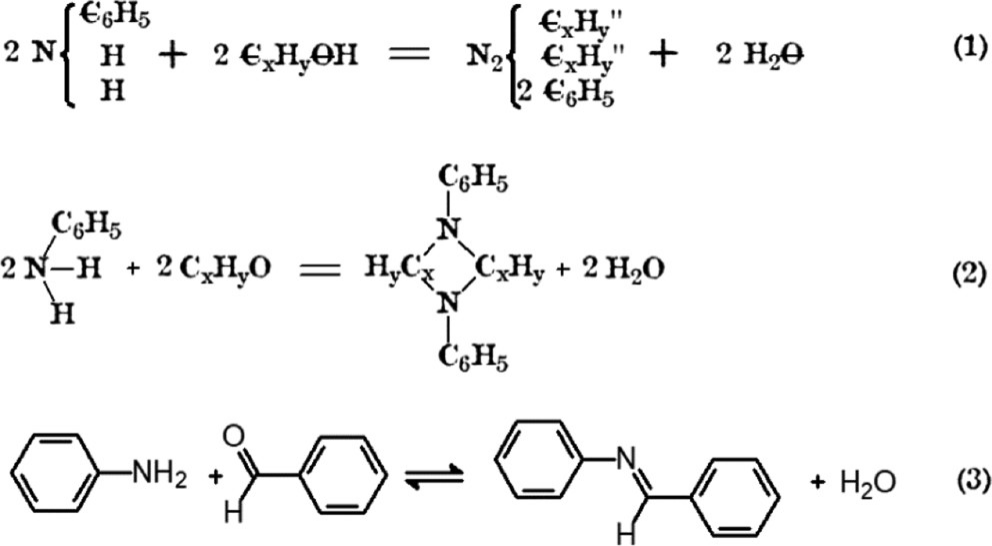
Reaction of aniline with an aldehyde (1) as depicted in Schiff’s 1864 article,^1^ (2) same reaction as 1, with the structural formulae of aniline and the Schiff base, and (3) reaction of aniline and benzaldehyde, illustrated according to the modern view. The reactions were drawn by the author using ancient and modern fonts.

In March 1864, Hugo Schiff (30), an assistant at the Chair of Chemistry at the University of Pisa, held by Professor Paolo Tassinari (1829–1909), submitted to *Annalen der Chemie und Pharmacie* a paper entitled “Mittheilungen aus dem Universitäts-laboratorium in Pisa” (Communications from the University Laboratory in Pisa).^1^ He was about to leave Pisa for the Regio Istituto di Studi Superiori Pratici e di Perfezionamento (Royal Institute for Practical and Advanced Studies) in Florence, where he had been nominated Professor of Chemistry, the first in the Institute. In the article, Schiff provided an account of the research work that he had carried out during his one year stay in Pisa. The paper (seven and one-half pages) consists of two sections: one (six pages) reported on quinolin and on its metal derivatives (Zn, Hg, Sb, and Bi), “Untersuchungen über das Chinolin” (Investigations on Quinolin), and the second section, one and a half pages, “Eine neue Reihe organischer Basen” (A new series of organic bases), described the reactions of aniline with aldehydes. Figure 1 shows a chemical equation directly taken from the paper, in which 2 mol of aniline reacts with 2 mol of an aldehyde, whether aliphatic or aromatic, to give 1 mol of base and 2 mol of water.

**Figure 2:**
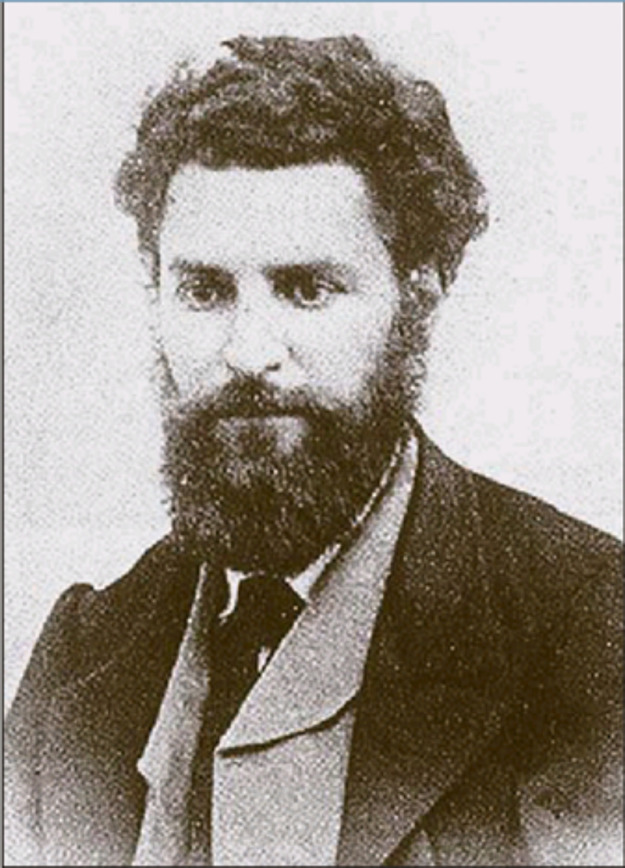
Picture of Hugo Schiff (Frankfurt am Main, 26 April 1834–Florence, 8 September 1915), taken in 1860. In that year Schiff, a Privatdozent at the University of Bern, attended the Karlsruhe Congress (3–5 September 1860), the first international conference of chemistry worldwide. Public domain image; source: http://www.biospektrum.de/blatt/d_bs_pdf&_id=932204.

The [2 + 2] stoichiometry of reaction 1 is surprising to modern chemists, who correctly interpret Schiff base condensation according to reaction 3, in which aniline and benzaldehyde react according to a [1 + 1] stoichiometry to give an imine derivative and a water molecule. In a later paper,^2^ Schiff provided the structural formula of the product of the condensation, as illustrated in reaction 2. In particular, Schiff hypothesized for the base a cyclic structure to allow nitrogen to achieve its typical valence. Schiff did not know the double bond and could not envision the formation of the imine bond. Moreover, it should be noted that in the original equation (reaction 1), both C and O symbols are barred by a short horizontal line, which conventionally indicates that this atom stands for two equivalents (O, 2 × 8; C, 2 × 6). At the Karlsruhe Congress (3–5 September 1860), Stanislao Cannizzaro (1826–1910) proposed a new scale of atomic weights, based on the assumption that hydrogen exists as a diatomic molecule (H_2_) and possesses a molecular weight = 2.00.^3^ Cannizzaro’s scale was adopted by most of the chemists, and bar convention persisted for a few more years, whereupon the bars were dropped. Schiff, aged 26 (Figure 2), participated in the Karlsruhe Congress but in 1864 still adhered to the bar convention. In Karlsruhe, Schiff met Cannizzaro, with whom he later collaborated in giving a sound and well-defined framework to Italian chemistry and founded the first Italian chemical magazine (*Gazzetta Chimica Italiana*, 1870). Among the 127 attendants of the Congress, German chemists represented the most numerous group (57) followed by chemists from France (21) and from United Kingdom (18). Quite curiously, Schiff was not considered a member of the German team but of the Swiss one (6) because at that time, he was a Privatdozent at the University of Bern.

Settled in Florence, at the end of December 1864, Schiff submitted to *Annalen* the complete paper (Eine neue Reihe organischer Diamine, 28 pages)^4^ followed by a paper on the same topic (Eine neue Reihe organischer Diamine – Zweite Abtheilung, 45 pages),^5^ submitted on April 1866. Still, in 1866, Schiff published an Italian version of his studies on the same topic (“Sopra una nova serie di basi organiche” [On a new series of organic bases])^2^ in a scientific-economic magazine published in Palermo (*Giornale di scienze naturali ed economiche*). At that time, Cannizzaro, a professor at the University of Palermo, used to publish some of his papers in *Giornale* and may have stimulated his colleague and friend Schiff to submit articles. Quite interestingly, in the paper, bars dropped from C and O symbols, perhaps because Schiff wanted to please his friend Stanislao, more probably because the local typographer did not have available such typefaces. Noticeably, in the same issue, there was also an inorganic paper by Schiff, “Cenni di chimica mineralogica” (Elements of Mineralogical Chemistry), which demonstrated the versatility and the variety of interests of the author. After the 1864–1866 period, Schiff, eager to explore new and unknown fields of organic and inorganic chemistry, was not any longer interested on his bases. Nevertheless, he had sown a precious seed from which a vigorous plant grew and is still growing.

## 2 Schiff Bases and Coordination Chemistry

**Figure 3:**
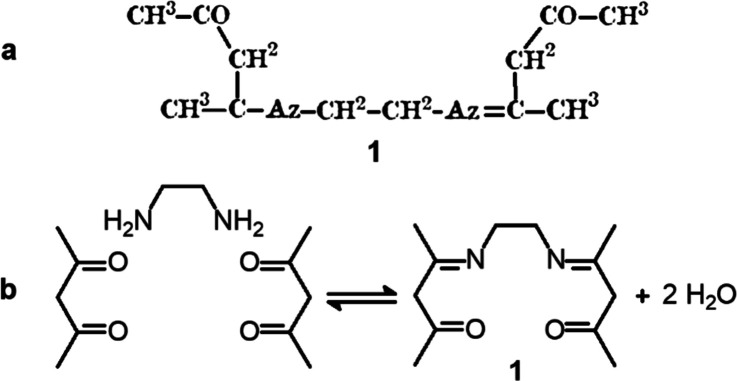
(a) Formula of the product of the reaction of ethylenediamine (1 equiv) and acetylacetone (2 equiv), as drawn by Combes.^6^ (b) Schiff base condensation written according to the modern view. The reactions were drawn by the author using ancient and modern fonts.

Schiff bases are classical ligands for metal ions of p, d, and f blocks, which have significantly contributed to the development of coordination chemistry on both basic and applicative aspects, with a special reference to catalysis. The first metal complex of a Schiff base was synthesized by Alphonse Combes (1854–1907), a professor of Industrial Chemistry at the École Municipale de Physique et de Chimie in Paris.^6^ Combes in 1889 made ethylenediamine react with two equivalents of acetylacetone, as pure substances. A highly exothermic reaction took place, with formation of water and, on cooling, precipitation of a white crystalline mass (m.p. of 111 °C). Figure 3 shows the structural formula of the product as drawn by Combes (a) compared with that outlined in a modern style (b).

At that time, multiple bonds were already known to chemists, and Combes wrote correctly the formula of the product (**1** in Figure 3a). The reaction is a classical Schiff base condensation (but Schiff was not cited in the article) involving two ketonic carbonyl groups and two primary amine groups, with formation of two imine bonds and elimination of two water molecules. Combes disregarded the current accepted nomenclature and used for nitrogen the symbol Az (from azote), introduced by Lavoisier in 1772. He also indicated the number of a given atom in a formula with a superscript, definitively replaced by a subscript in a short time.

**Figure 4:**
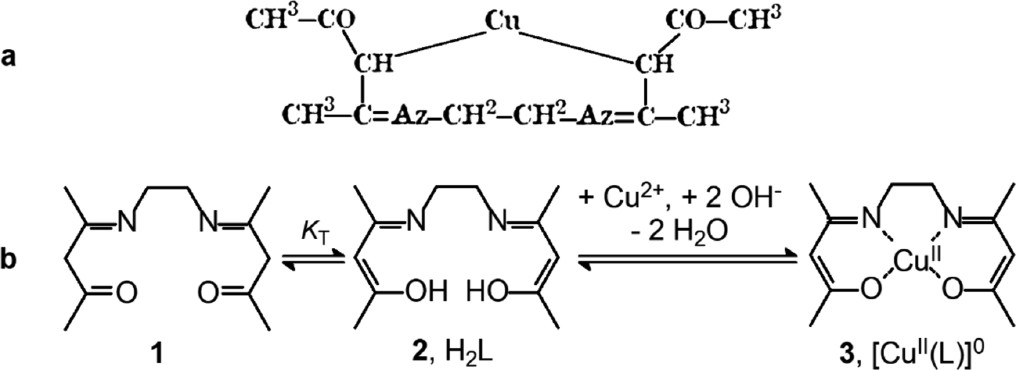
(a) Structural formula proposed by Combes for the neutral complex obtained through the reaction of copper(II) acetate with Schiff base **1**, shown in Figure 3a.^6^ (b) Schiff base **1** (keto form) undergoing a tautomeric equilibrium of constant *K*_T_ to give enol form **2**. In the presence of a base (e.g., acetate), **2** (H_2_L) deprotonates, and the enolate ion L^2–^ forms stable neutral complex **3**, [Cu^II^(L)]. The reactions were drawn by the author using ancient and modern fonts.

Then, on treating an aqueous solution of white product **1** with an aqueous solution of copper(II) acetate, a nice violet precipitate was obtained, in the form of thin plates, insoluble in water, and fairly soluble in ethanol and chloroform. On the basis of the gravimetric analysis of copper(II), Combes suggested the molecular formula C_12_H_18_N_2_O_2_Cu (right) and the structural formula shown in Figure 4a (wrong).

Combes was aware of the acidic nature of the −CH_2_– groups linked to the carbonyl group and to the imine group and correctly hypothesized their deprotonation in the presence of a base (e.g., acetate) but did not know the existence of the keto–enol tautomerism (Figure 4b). Thus, he hypothesized the formation of Cu^II^–C bonds. The monumental work by Alfred Werner (1866–1919) on metal coordination chemistry would be published four years later,^7^ and Combes could not know that transition metal ions do not have any affinity toward carbon donor atoms but are eager to interact with nitrogen donor atoms and oxygen donor atoms especially if detaining a formal negative charge.

**Figure 5:**
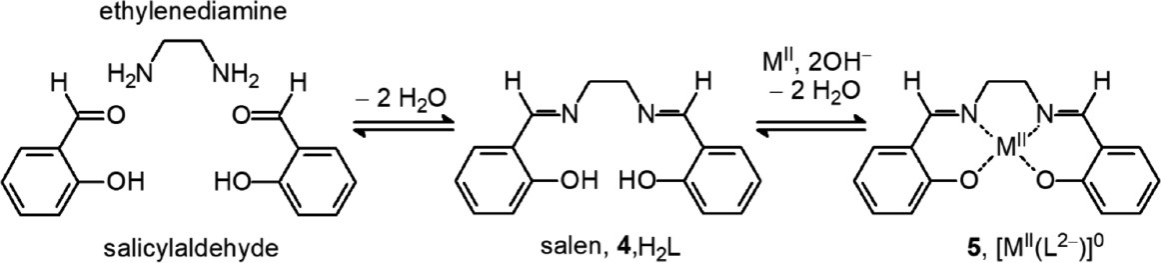
Synthesis of salen (**4**) through Schiff base condensation of ethylenediamine and salycylaldehyde. In a basic solution, salen releases two hydrogen ions and chelates a divalent metal ion to give a stable neutral complex (**5**, [Co^II^(L^2–^)^0^]). Tsumaki in 1938 observed that [Co^II^(L^2–^)] is able to bind reversibly a dioxygen molecule, the first example of an artificial O_2_ carrier,^9^ thus opening one of the most intensively cultivated fields of coordination chemistry.

Enol form **2** in Figure 4b is a close relative of salen (**4**, in Figure 5), a classic ligand of transition and post-transition metals and a major player on the coordination chemistry stage.^8^

**Figure 6:**
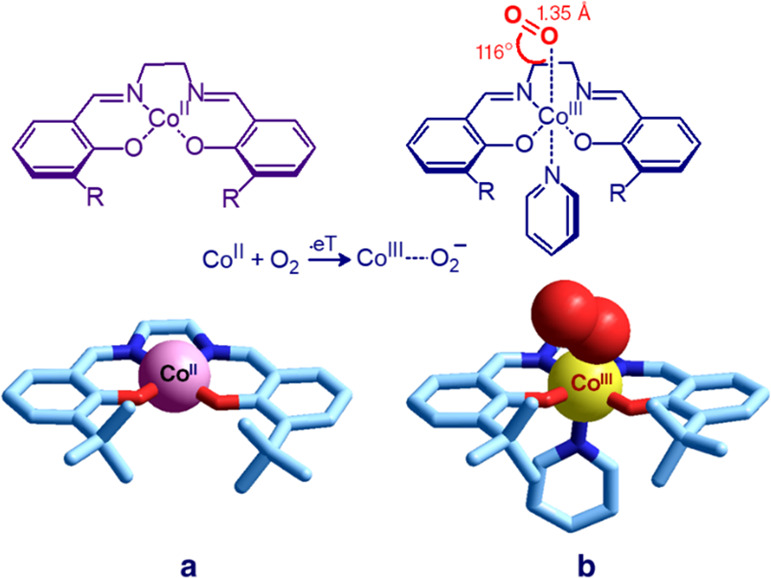
Crystal structures of cobalt salen complexes: (**a**) *N*,*N*′-ethylene-bis(3-*tert*-butylsalicylideneiminato)-cobalt(II)^10^ and (**b**) dioxygen-(*N*-pyridine)-*N*,*N*′-ethylene-bis(3-*tert*-butylsalicylideneiminato)-cobalt(III).^10^ In pyridine solution, O_2_ oxidizes Co^II^ to Co^III^ and is reduced to O_2_^–^ (superoxide). The process is described by the following equation: [Co^II^(salen)] + py + O_2_ ⇆ [Co^III^(salen)(py)(O_2_^–^)].

**Salen** is obtained by Schiff base condensation of 2 equiv of **sal**ycylaldehyde and 1 equiv of ethylenediamine (**en**). On addition of divalent metal acetate, the two phenolic −OH of H_2_L deprotonate, and L^2–^ forms a neutral complex with M^II^ ([M^II^(L^2–^)]^0^, **5**). The complex is strongly stabilized by the chelate effect. Cobalt(II) complexes of salen derivatives were the first synthetic complexes capable of absorbing and releasing reversibly dioxygen,^9^ thus mimicking natural oxygen carriers and storage proteins containing a transition metal, to which the oxygen reversibly coordinates: iron (Fe^II^/Fe^III^: myoglobin and hemoglobin) and copper (Cu^I^/Cu^II^: hemocyanin). Figure 6 shows the crystal structures of a cobalt(II) salen complex and of its oxygenated form.^10^

At room temperature, a red-brown square planar cobalt(II) complex (**a** in Figure 6), dissolved in pyridine, absorbs dioxygen to give dark brown complex **b**. The process involves an electron transfer from Co^II^ to O_2_, and **b** must be correctly described as a [Co^III^(salen)(py)(O_2_^–^)], in which the superoxide anion is bound to Co^III^. The system absorbs O_2_ at room temperature and releases it at higher temperature. This cycle may be repeated many times, although the activity of the complex toward dioxygen uptake slowly decreases on continued cycling, owing to decomposition.^10^

## 3 The Paradoxical Nature of the C=N Bond, Both Strong and Labile

**Figure 7:**
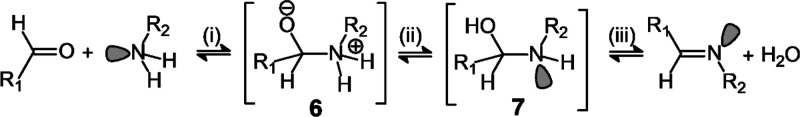
Mechanism of the reaction of the carbonyl group of an aldehyde with a primary amine operating in Schiff base condensations. The process is constituted by three reversible steps.

Figure 7 illustrates the mechanism of the reaction of the carbonyl group of an aldehyde with a primary amine.

The reaction proceeds through three reversible steps: (i) nucleophilic attack by the amine nitrogen atom to the carbonyl carbon atom, to give separated charge intermediate **6**; (ii) intramolecular proton transfer from the ammonium group to the carbinolate group to give carbinolamine **7**; (iii) water elimination and imine formation. The entire process is fully reversible. It derives that the C=N bond, strong as expected for a double covalent bond (C=N bond energy of 615 kJ mol^–1^), is subject to hydrolysis according to the reverse equilibrium in Figure 7, and its formation is therefore thermodynamically controlled. Covalent bonds in organic substances are typically slow both to form and to break. Thus, it is inert and irreversible, and its formation occurs under a kinetic control. On the other hand, there exists in nature a variety of reversible bonding interactions, e.g., hydrogen bonds: they are weak, reversible, fast to form, fast to break and operate under a thermodynamic control. The imine bond is unique: it is strong (it is a covalent bond and, what is more, double) and labile (like the hydrogen bond). Such a dual feature allows the synthesis of complex molecular systems from aldehydes and primary amines in a one-pot procedure: the reactants, put all together in the same vessel, undergo an unlimited sequence of fast and reversible attempts to finally give the desired product in good yield, as long as it is thermodynamically stable.

**Figure 8:**
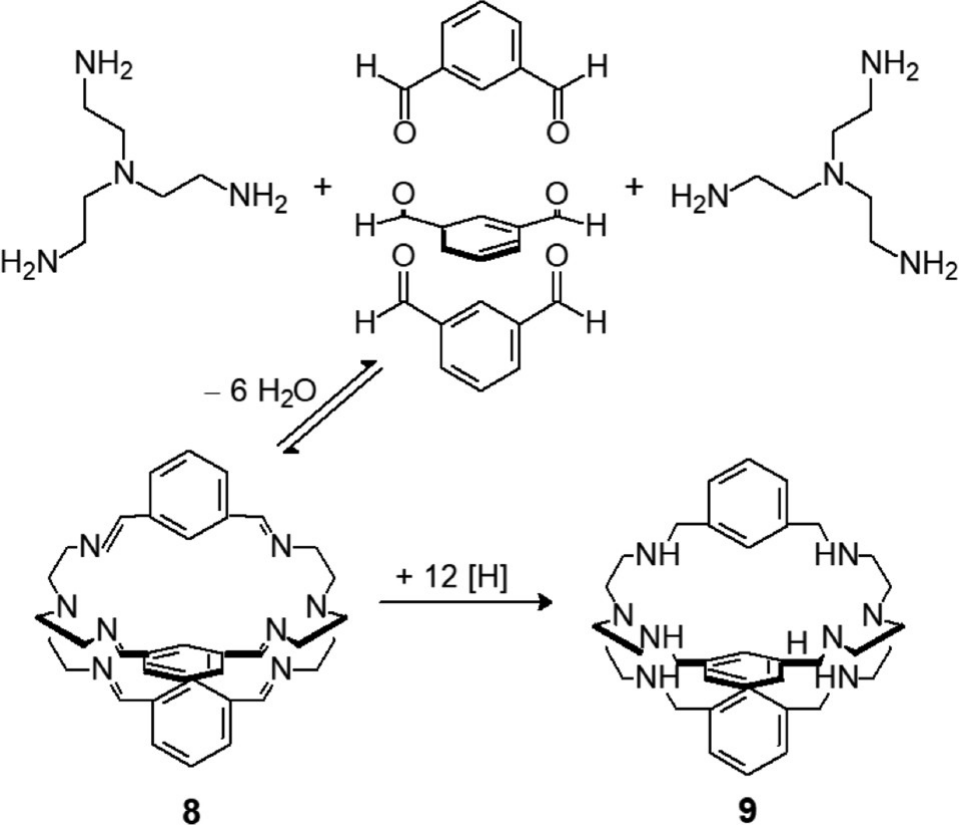
Schiff base condensation of two molecules of branched tetramine tren with three molecules of 1,3-benzene-dicarbaldehyde, to give unsaturated cage-shaped molecule **8**, whose six C=N bonds are subject to hydrolysis. Imine bonds are then hydrogenated to give kinetically stable octamine **9**, bistren.

A convincing example is provided by the reaction shown in Figure 8, leading to a cage-shaped macrobicyclic compound.

Two molecules of branched tetramine tren are allowed to react with three molecules of 1,3-benzene-dicarbaldehyde in methanol at room temperature. After a few minutes, Schiff base **8** precipitates as a white product. Such a product is not definitively stable, as it is subject to the reverse equilibrium, which restores the reactants, for instance, on addition of an acid. However, the six vulnerable C=N bonds can be “immobilized” through hydrogenation with NaBH_4_ to give cage-shaped macrobicyclic tetramine **9**. C–N bonds are weaker than corresponding C=N bonds (C–N bond energy of 290 kJ mol^–1^) but are inert and not prone to hydrolysis. In fact, cage-shaped octamine is stable both in strongly acidic and in strongly basic solutions. That as many as five particles spontaneously organized to give a complex structure is due to the reversible nature of the imine bonds. C=N bonds form (through Schiff base condensation) and break (through hydrolysis) unceasingly and quickly until, through a trial-and-error mechanism, the most thermodynamically stable structure is obtained: the hexaimine cage. The reason of such stability is not straightforward: it is possible that benzene residues in the cage are less exposed to the protic medium (MeOH) than in the dialdehyde, thus exerting a lower disturbing effect on solvent’s aggregation, which displaces to the right the condensation equilibrium.

**Figure 9:**
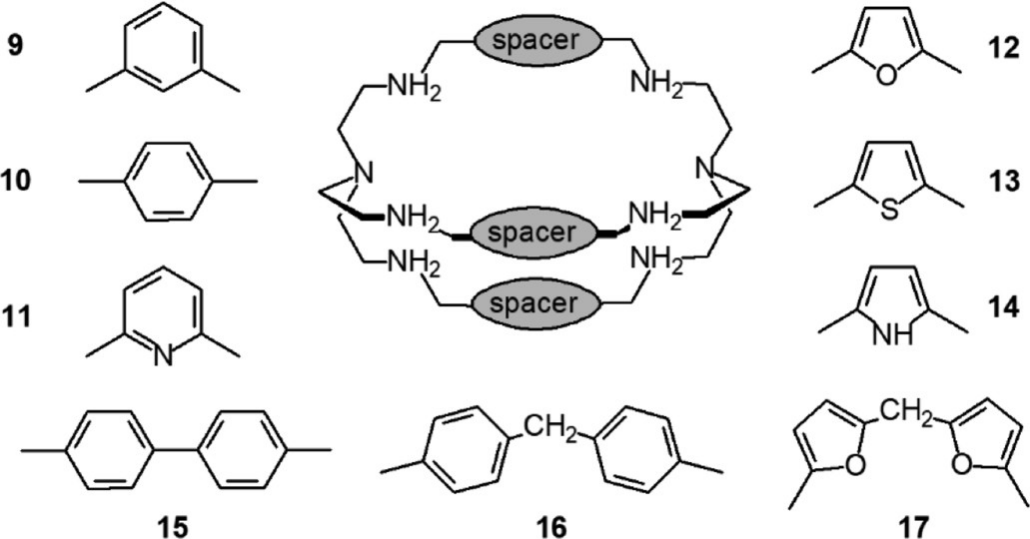
Bistren cages with varying spacers. Each spacer derives from the aldehyde used in the Schiff base condensation and defines the size of the cavity.

The size of the cavity of the bistren cage can be modulated at will by choosing the appropriate dialdehyde, which generates the appropriate spacer. A series of spacers used in the synthesis of bistren cages is shown in Figure 9.^11−14^

## 4 Bistrens: Comfortable Cages for Anions

**Figure 10:**
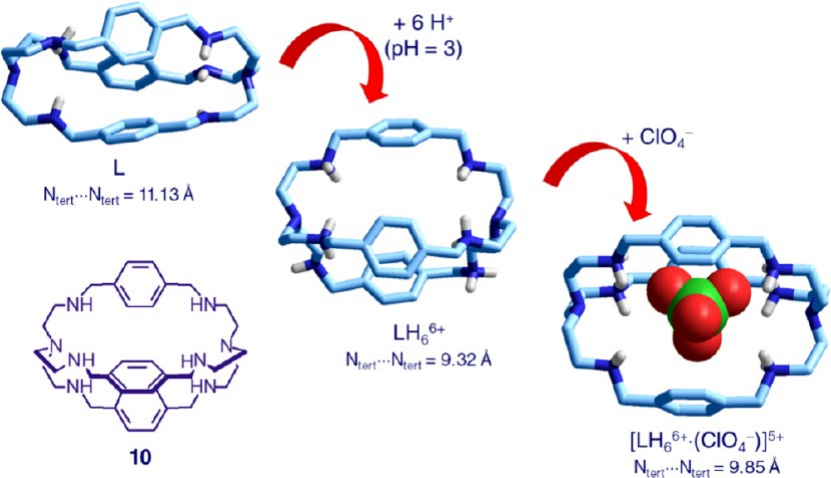
Cascade mechanism for the inclusion of ClO_4_^–^ into the hexaprotonated form (LH_6_^6+^) of bistren **10**, to give the inclusion complex [LH_6_^6+^·(ClO_4_^–^)]^5+^. C–H hydrogens, solvating molecules, and counteranions have been omitted for clarity.

Bistren cages can act as containers of anions of varying sizes and shapes. However, if you wish to include a negatively charged particle, you must make the shelter appropriate for it, for instance, creating a positive charge inside. In particular, in aqueous solution adjusted to pH 3, the six secondary nitrogen atoms of bistren are protonated, and the two pivot tertiary amine groups are not. At this stage, the hexammonium receptor can accommodate the anion, provided that there exists steric complementarity between the hosting cavity and the guest. The process takes place in two steps, as illustrated by a cascade diagram illustrated in Figure 10, in which the hexaprotonated form of bistren **10** incorporates the ClO_4_^–^ anion.

Single crystal X-ray diffraction studies have shown that void octamine L (**10**) has a rather elongated ellipsoidal shape, with a distance between the two tertiary nitrogen atoms N_tert_···N_tert_ = 11.13 Å.^15^ On protonation, to give LH_6_^6+^, the reciprocal electrostatic repulsions between the six ammonium groups forces the framework to assume a spheroidal shape, with a substantial reduction of the N_tert_···N_tert_ distance (9.32 Å).^16^ On inclusion of ClO_4_^–^, to form the inclusion complex [LH_6_^6+^·(ClO_4_^–^)]^5+^, such a distance does not change very much (N_tert_···N_tert_ = 9.85 Å), thus maintaining its spheroidal shape.^17^ However, the framework readjusts to point the N–H fragments of the ammonium groups toward the oxygen atoms of perchlorate. The establishing of electrostatic interactions and of hydrogen bonds between the highly polarized N–H fragments and perchlorate oxygen atoms are responsible for the stability of the complex both in acidic aqueous solution and in the solid state. LH_6_^6+^ (L = **10**) shows a special affinity toward tetraoxo anions and forms stable inclusion complexes with SO_4_^2–^, SeO_4_^2–^, ReO_4_^–^, and TcO_4_^–^ and, in addition, with tetrahedral anion S_2_O_3_^2–^.^18^ In all the anion inclusion complexes, the hexammonium receptor exhibits a spheroidal shape with an N_tert_···N_tert_ distance of ca. 10 Å.

**Figure 11:**
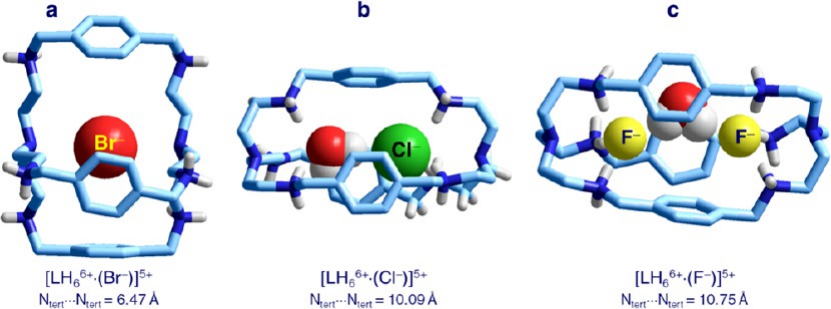
Crystal structures of the inclusion complexes of LH_6_^6+^ (L = **10**) with (a) Br^–^,^19^ (b) Cl^–^,^20^ and (c) F^–^.^21^

However, LH_6_^6+^ (L = **10**) possesses a rather flexible framework and is able to shrink its cavity enough to incorporate a monoatomic anion. This is the case for the bromide inclusion complex, whose structure is shown in Figure 11a.^19^

In order to establish strong electrostatic and hydrogen bonding interactions with Br^–^ (ionic radius of 1.96 Å), the hexammonium cage shrinks its cavity to a very short N_tert_···N_tert_ distance of 6.47 Å. The inclusion of the smaller Cl^–^ ion (ionic radius of 1.81 Å) would require a further contraction of the cavity, which would involve a too high energy cost. Thus, in order to fit the cavity in its relaxed conformation, chloride enters the cage accompanied by a water molecule (see Figure 11b).^20^ Each guest occupies and interacts with a trenH_3_^3+^ subunit. H_2_O is a cooperative guest, which receives six H-bonds from facing N–H fragments but also donates an H-bond from one of its O–H fragments to the close Cl^–^ anion. Moreover, in the case of the smallest fluoride ion (ionic radius of 1.28 Å), in the relaxed hexammonium cage, there is room for two F^–^ anions bridged by a water molecule (see Figure 11c).^21^ H_2_O is there to fill the cavity and to shield the electrostatic repulsions between the two fluoride ions. Thus, hexaprotonated bistren cages are versatile anion receptors, which are capable to rearrange their framework to fulfill guests’ geometrical requirements. They do not show any size and shape selectivity in anion inclusion, a behavior that can be ascribed to the intrinsic weakness and poor directionality of electrostatic and hydrogen bonding interactions.

**Figure 12:**
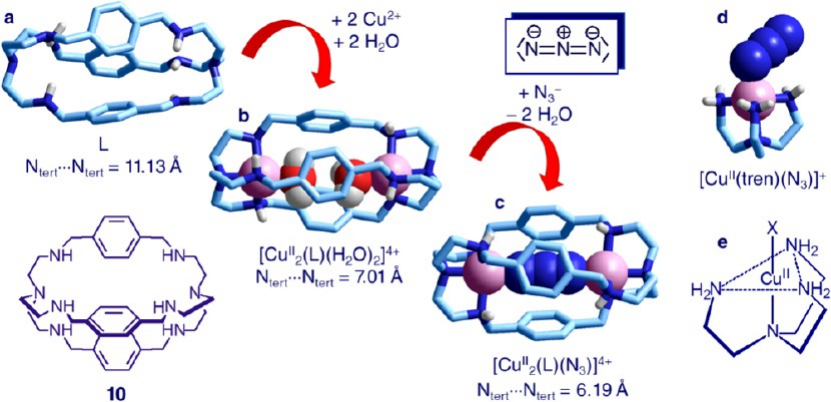
Cascade mechanism for the formation of the ternary dinuclear complex [Cu^II^_2_(L)(N_3_)]^3+^ (L = **10**, a). The azide ion displacing the two metal-bound water molecules in the [Cu^II^_2_(L)(H_2_O)_2_]^4+^ complex (b)^22^ and bridging the two Cu^II^ ions to give [Cu^II^_2_(L)(N_3_)]^3+^ (c).^23^ (d) Structure of the [Cu^II^(tren)(N_3_)]^+^ complex,^24^ showing the bent coordination mode of N_3_^–^ (Cu^II^–N–N angle = 116°), ascribed to the sp^2^ hybridization of metal-bound nitrogen atoms. (e) Trigonal bipyramidal geometry of a [Cu^II^(tren)X] + complex (X^–^ = mononegative anion).

There exists another way to make the bistren cavity appropriate for anions: putting in the cage two transition metal ions, each interacting with one bistren subunit. Between the two metals, there is room for an ambidentate anion capable to act as a bridge. Moreover, the intermetallic distance can be modulated by varying the length of the spacer, which may generate selectivity in anion inclusion and recognition. Again, we are in the presence of a cascade process, which is illustrated in Figure 12.^18^

In the first step, in an aqueous solution of a transition metal salt, e.g., Cu(ClO_4_)_2_, octamine cage **10** uptakes two Cu^II^ ions. Each metal ion goes to occupy a tren subunit. Copper(II) complexes of branched tetramine tren typically show a trigonal bipyramidal geometry, with the primary nitrogen atoms of tetramine spanning the three equatorial positions and the tertiary one positioned in one axial position. The remaining axial site is occupied by a fifth donor atom of an exotic ligand (see Figure 12e). In particular, in the dimetallic complex in water, the two available axial positions are occupied by two H_2_O molecules (Figure 12b). Such a coordinative arrangement has been observed in the solid state.^22^ Then, on addition of a polyatomic ambidentate ligand, e.g., azide, the two water molecules are displaced and replaced by the two terminal nitrogen atoms of the anion, to give the ternary complex [Cu^II^_2_(L)(N_3_)]^3+^ (Figure 12c).^23^ The azide ion, which in the [Cu^II^(tren)(N_3_)]^+^ complex exhibits a bent coordination mode (Cu–N–N angle = 116.2°),^24^ due to the sp^3^ hybridization of the terminal nitrogen atoms (see Figure 12d), in the dimetallic complex is sterically forced to be collinear with the two Cu^II^ ions as well as with the two nitrogen tertiary atoms of bistren.

**Figure 13:**
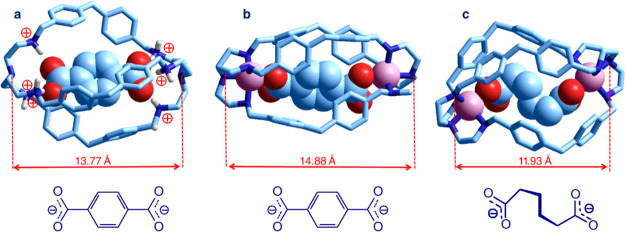
Crystal structures of dicarboxylate inclusion complexes by receptors derived from bistren **10** (= L): (a) [LH_6_·(terephthalate)]^4+^.^25^ (b) [Cu^II^_2_L·(terephthalate)]^2+^.^26^ (c) [Cu^II^_2_L·(adipate)]^2+^.^26^

Bistrens with larger spacers can include large organic anions. For instance, the hexaprotonated LH_6_^6+^ and the dicopper(II) [Cu^II^_2_(L)]^4+^ derivatives of **16** incorporate selectively aromatic and linear aliphatic dicarboxylates of varying lengths. Figure 13a,b shows the crystal structures of the inclusion complexes of LH_6_^6+^ with terephthalate^25^ and of [Cu^II^_2_(L)]^4+^ with terephthalate (a and b) and adipate (Figure 13c).^26^

In all the complexes, the cage undergoes a significant conformational rearrangement to afford the formation of the strongest interactions, whether electrostatic/hydrogen bonding or metal–ligand. A major rearrangement is observed in the [Cu^II^_2_L·(adipate)]^2+^ complex, which exhibits the shortest N_tert_···N_tert_ distance and shows a spheroidal shape.

**Figure 14:**
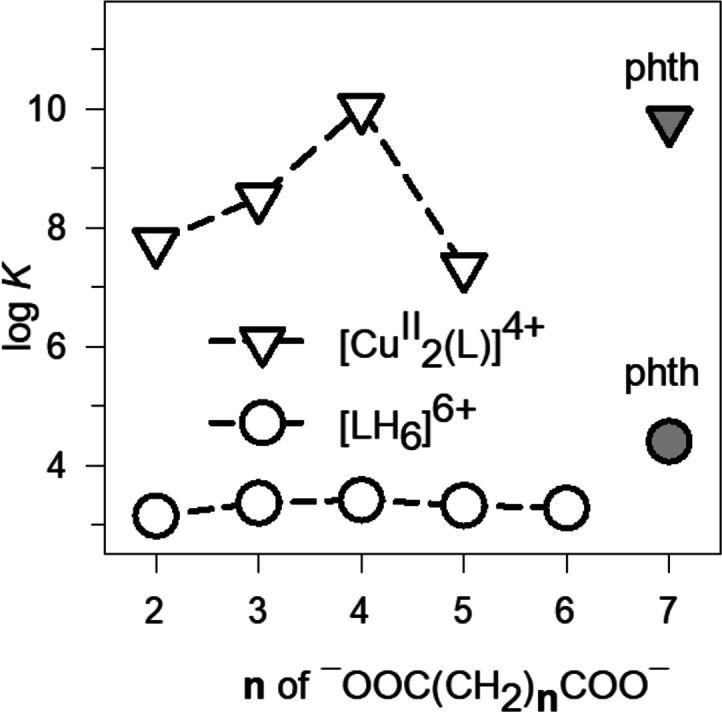
L = **16**. Plot of the log *K* values vs. the number of methylene groups *n* of the α,ω-dicarboxylic acids of formula ^–^OOC(CH_2_)*_n_*COO^–^ (A^2–^). The log *K* values refer to the equilibria: (i) empty circles, LH_6_^6+^ + A^2–^ ⇄ [LH_6_···A]^4+^ (L = **6**), pH 6, 20 °C; (ii) empty triangles, [Cu^II^_2_(L)]^4+^ + A^2–^ ⇄ [Cu^II^_2_(L)(A)]^2+^, 50/50 water/ethanol, pH 7.2, 25 °C. Full symbols indicate log *K* values for the inclusion of terephthalate by [Cu^II^_2_(L)]^4+^ (triangle) and by A^2−^ (circle).

Equilibrium studies in an aqueous solution buffered at pH = 6, with pyridine + CF_3_COOD, 10^–2^ M, showed that log *K* values for the inclusion by LH_6_^6+^ of linear aliphatic carboxylates of formula ^–^OOC–(CH_2_)*_n_*–COO^–^ (*n* = 2–6) are nearly the same, indicating a lack of inclusion selectivity (see open circles in Figure 14).^25^ On the other hand, equilibrium studies in a 50/50 water/ethanol (v/v) solution showed that the dimetallic receptor [Cu^II^_2_(L)]^4+^ exerts a well-defined inclusion selectivity for adipate (*n* = 4). Linear aliphatic dicarboxylates of lower and higher length do not fit so well with the receptor’s intermetallic distance and show inclusion constants of 2–3 orders of magnitude smaller (see open triangles in Figure 14).^26^

**Figure 15:**
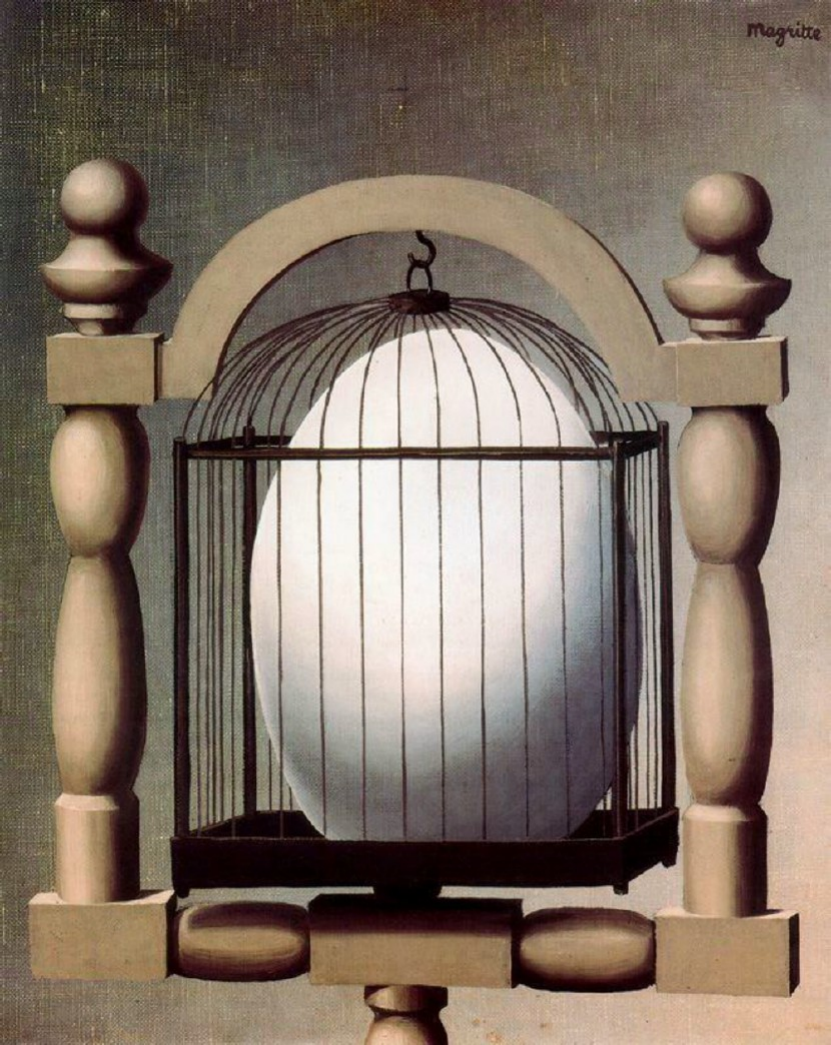
René Magritte’s *Elective Affinities* (1933), oil on canvas (41 cm × 33 cm). Private collection. Public domain image; source: https://www.renemagritte.org/elective-affinities.jsp.

Chemists are often inclined to give molecular systems names borrowed from everyday life. Bistren receptors belong to the family of cages. Cages are familiar objects for the human kind since the Neolithic Age. Since then, humans have built and used cages but did not love them for several reasons: (i) cages are a symbol of forced constriction and freedom deprivation for living beings, (ii) they exhibit to the public private details of the imprisoned individual, affirming its state of weakness and dependence, (iii) typical guests of cages are tender and undefended beings (a canary or a parrot). Humans use cages mostly for leisure but are not proud of this practice and are reluctant to its emphasis. It is probably for this reason that artists have not been inspired by cages, in both painting and handicraft. There are probably only two relevant paintings featuring a cage, and they are quite recent on the timescale of art history. One has been painted by René Magritte (1898–1967)—*Elective Affinities*, 1933, shown in Figure 15. The prisoner of the cage is an egg, and probably, the artist intended to bewilder the viewer by illustrating the delayed affinity of two objects to each other, the cage and the egg, from which the typical guest of the cage, a bird, originates. Perhaps, the painting intends to communicate a distrustful message: every human, even before his birth, is destined to live within the narrow limits and the severe rules of a merciless society.

Fortunately, in chemistry, things are different: synthesizing a molecular cage and confining in it a chemical species (a metal ion, an anion, or a molecule) are considered a deserving and admirable action. The design and synthesis of cages at a molecular level have become so popular and distinguished, an activity mentioned in the Merriam–Webster dictionary: “an arrangement of atoms or molecules so bonded as to enclose a space in which another atom or ion (as of a metal) can reside”.^27^ However, the analogy between the cages of the macroscopic world and those of the molecular world may not be fully justified. In fact, a living being in a macroscopic cage stays under an unpleasant kinetic control: it may have the greatest tendency to escape from the cage, but this event is prevented by an insurmountable activation barrier (dense metal bars and a firmly locked gate). On the molecular side, such a kinetically controlled situation is rarely observed. We have seen that as far as anions are concerned, no kinetic barrier exists for getting in/out of a bistren cage: the host is no longer a prisoner, and its stay in the cage is thermodynamically controlled.

A second example of cages in figurative arts is provided by the famous and intriguing wood-engraved print by M. C. Escher (*Stars*, 1948, https://mcescher.com/lw-359/). The print depicts two chameleons confined in a cage composed of three interlocking regular octahedra, floating through space. The image illustrates the attempt of the universe to impose its immutable and celestial order (represented by polyhedra) to the overwhelming force of life (the two chameleons).

**Figure 16:**
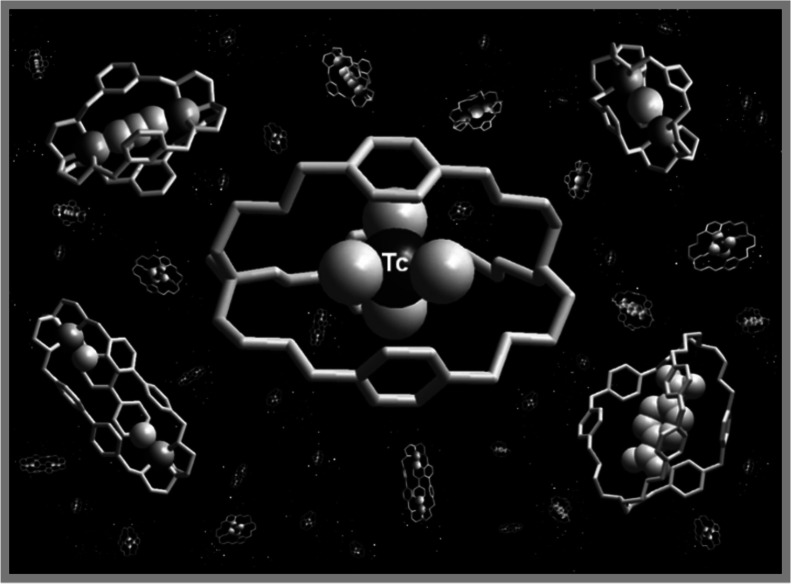
Chemical imitation of Escher’s print “*Stars*” illustrating a variety of anions caged by bistren receptors. This image appears as the cover for the March 28, 2015 issue of *Organic & Biomolecular Chemistry*, (Volume 13, Number 12) to accompany an article by the author. Adapted from Fabbrizzi et al. (2015). Copyright 2015 Royal Society of Chemistry.

A chemical imitation of Escher’s print is shown in Figure 16, illustrating a variety of anions caged by bistren receptors. The artistic quality of the picture is rather poor, but the drawn chemical cages remain fascinating and promising objects, deserving attention and further investigations in their basic and applicative aspects.

## 5 Schiff Base Condensations Driven by Metal Ions: Rings and Macrocycles

**Figure 17:**
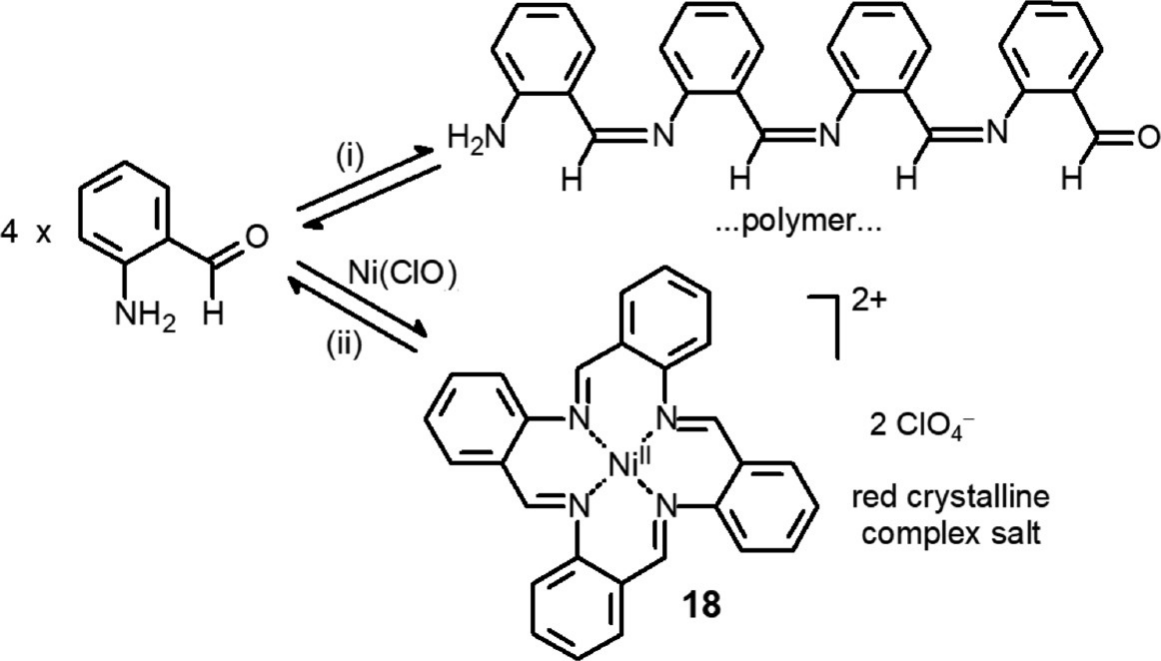
Schiff base self-condensation of *o*-aminobenzaldehyde in the absence (route (i)) and in the presence of a nickel(II) salt (route (ii)). The Ni^II^ ion acts as a square template and drives the condensation to the formation of a tetra-aza macrocycle.^30^

Transition metal ions can address Schiff base condensations to sophisticated shapes acting as templates. An example is provided by the self-reaction of *o*-aminobenzaldehyde, illustrated in Figure 17.

The amine group and the aldehyde group of the same molecule are sterically prevented to give intramolecular Schiff base condensation. Thus, head–tail intermolecular reactions take place to give a linear polymer in the form of a sticky yellow product, route (i) in Figure 17. On the other hand, if nickel(II) perchlorate is added to a refluxing ethanolic solution of *o*-benzaldehyde in the stoichiometric ratio 1:4, a red crystalline precipitate forms, consisting of the perchlorate salt of a Ni^II^ complex (**18**) of a tetra-aza macrocycle named TAAB (acronym of **T**etr**A**-**A**mino-**B**enzaldehyde), route (ii) in Figure 17.^28^ Formation of the macrocycle is driven by Ni^II^, which, as a d^8^ cation, favors square coordination and addresses Schiff base condensation in such a way to be coordinated by four imine nitrogen atoms positioned at the corners of a square. Thus, Ni^II^ behaves as a square template. The [Ni^II^(TAAB)]^2+^ complex is exceptionally stable, suffering no decomposition in boiling concentrated HNO_3_, HCl, and HClO_4_. Hydrolysis of the imine bond is prevented by the closed structure of the macrocycle and by the protective effect of the coordination. The same template effect is exerted by Cu^II^, which shows a marked preference toward tetragonal coordination and may act as a square template. Under the previously described conditions, a dark green microcrystalline product is obtained, [Cu^II^(TAAB)](NO_3_)_2_.

**Figure 18:**
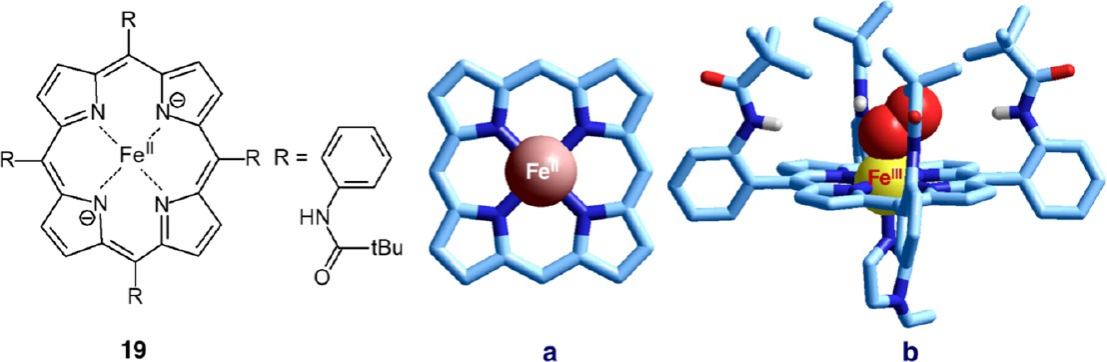
Synthetic porphyrin (**19**). (a) Crystal structure of the Fe^II^ complex with porphyrin **19**,^29^ from which 2-((*tert*-butyrylamino)amino)phenyl substituents have been removed (top view). (b) Crystal structure of the oxygenated form of complex **19**, which has to be considered a complex of Fe^III^, to which a superoxide ion (O_2_^–^) is axially coordinated according to a side-on bonding mode (Fe^III^–O–O angle = 130°) and the other axial position is occupied by 1-ethyl-imidazole.^31^

The structural formula of TAAB is highly symmetric and reminiscent of that of porphyrin. The Fe^II^ complex of porphyrin (heme) is an essential part of metalloproteins, in charge of fundamental functions (transport and storage of dioxygen, electron transfer in membranes, and elsewhere). The formula of synthetic porphyrin whose iron(II) complexes reversibly with dioxygen is shown in Figure 18 (**19**).

Figure 18a shows the crystal structure of the Fe^II^ complex of synthetic porphyrin without 2-((*tert*-butyrylamino)amino)phenyl substituents.^29^Figure 18b displays the oxygenated form of the same complex. On oxygenation, one electron is transferred from Fe^II^ to O_2_, and the product should be considered an Fe^III^–O_2_^–^ adduct. Extended delocalization of π electrons makes the porphyrinato ring almost perfectly planar. Such an aesthetically agreeable molecule is a sophisticated product of natural evolution dating back to 4 billion years.

**Figure 19:**
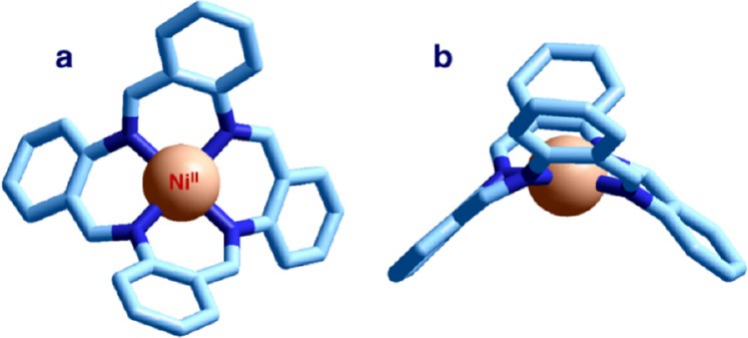
Crystal structure of the complex salt [Ni^II^(TAAB)(MeCN)_2_](I_3_)_2_^30^ (hydrogen atoms, metal-coordinated MeCN molecules, and I_3_^–^ counterions have been omitted for clarity). (a) Top view. (b) Lateral view.

The crystal structure of the [Ni^II^(TAAB)]^2+^ complex is not as nice and appealing (Figure 19).^30^

The arrangement of a TAAB macrocycle is anything but planar, a behavior ascribed to the absence of π-delocalization. In particular, the ligand adopts a saddle conformation. The old saying goes “don’t put the saddle before the horse”. This is what cultural evolution pretentiously did by synthesizing [Ni^II^(TAAB)]^2+^, but it failed miserably the competition with the more patient and wise natural evolution.

## A Tetrahedral Container for a Tetrahedral Molecule, P4

**Figure 20:**
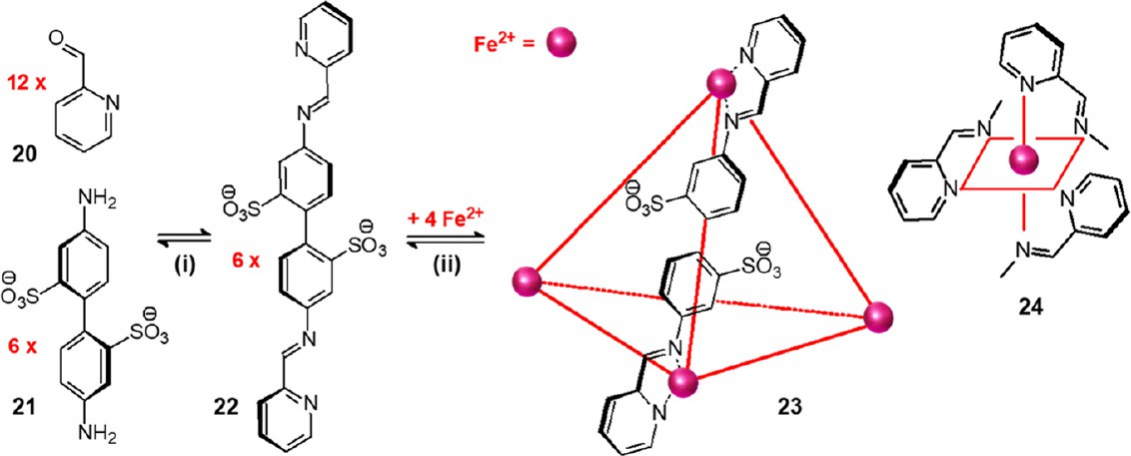
One-pot template synthesis of tetrahedral molecular system **23**.^33^ The process can be ideally divided into two separate steps: (i) Schiff base condensation of 12 mol of 2-formylpyridine (**20**) and of 4,4′-diaminobiphenyl-2,2′-disulfonate (**21**) to give **22** and (ii) binding of three imino-pyridine fragments from three distinct molecules of **22** to Fe^II^ according to an octahedral coordination geometry, which gives tetrahedrally shaped molecule **23**. Adapted from ref (31). Copyright 2008 Wiley–VCH Verlag GmbH & Co. KGaA.

Metal template Schiff base condensations may give rise to more sophisticated and aesthetically agreeable shapes than the uneven square described in the previous section. A good example is provided by a tetrahedron. Nitschke et al. synthesized a tetrahedral molecular system through a metal template Schiff base condensation process, as illustrated in Figure 20.^31^

**Figure 21:**
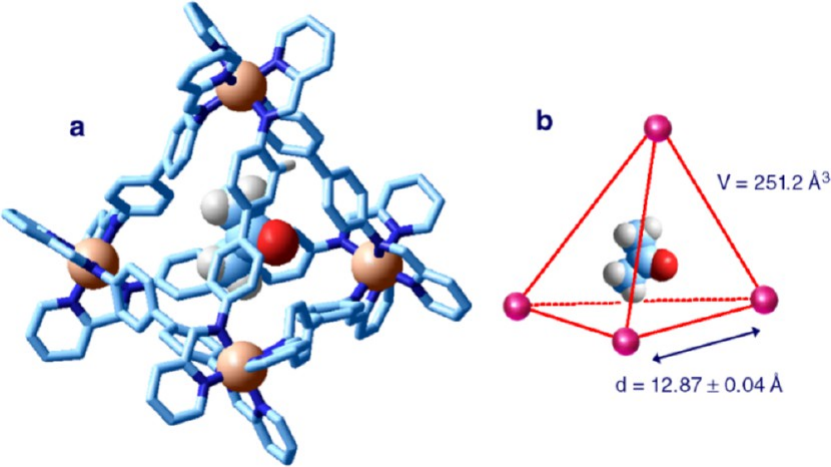
(a) Crystal structure of the salt (Me_4_N)_4_[Fe^II^_4_L_6_]·(CH_3_)_2_CO·H_2_O (L = **22**).^31^ Covalently linked sulfonate groups, hydrogen atoms, methylammonium ions, and solvating water molecules are omitted for clarity. An acetone molecule is well included in the tetrahedral receptor. (b) Sketch of the tetrahedron whose vertices are occupied by Fe^II^ ions. Adapted from ref (31). Copyright 2008 Wiley–VCH Verlag GmbH & Co. KGaA.

The synthesis, which proceeds according to a one-pot mode in aqueous solution at 50 °C, is split for clarity in Figure 20 in two consecutive equilibria: (i) Schiff base condensation of 12 mol of aldehyde **20** with 6 mol of dianiline **21** to give 6 mol of linear di-imine **22**. Each molecule of **22** contains at its ends two bidentate units N∩N, each one possessing one sp^2^ pyridine nitrogen atom and one sp^2^ imine nitrogen atom. Indeed, Fe^II^ (d^6^ electronic configuration) has been chosen as a templating ion because it shows a marked affinity toward sp^2^ nitrogen atoms, to give a six-coordinated complex of octahedral geometry (formula **24** in Figure 20). The only way for making Fe^II^ coordinated by three N^N subunits is that the six molecules of **22** position themselves along the six edges of a tetrahedron whose four vertices are occupied by Fe^II^ ions. This gives rise to tetrahedral molecular system **23**. The driving force of the process is the formation of four [Fe^II^(N∩N)_3_]^2+^ complex subunits made stable by N (sp^2^)–Fe^II^ (d^6^ low-spin) coordinative interactions, both σ and π in nature. Moreover, the intrinsic inertness of the [Fe^II^(N∩N)_3_]^2+^ subunit imparts kinetic stability. System **23** is an anion of charge 4⊖, which results from the balance of the 8⊕ charge of the 4 Fe^II^ ions and the 12⊖ charge of the 12 sulfonate groups, [Fe^II^_4_L_6_]^4–^ (L = **22**), and was isolated as a dark red methylammonium salt. On recrystallization from water/acetone, dark red crystals of a diamagnetic salt of formula (MeNH_3_)_4_[Fe^II^_4_L_6_]·(CH_3_)_2_CO·H_2_O (L = **22**) suitable for X-ray diffraction studies were obtained. Tetramethyammonium was present because disulfonate **21** had been obtained in situ by neutralization of the corresponding disulfonic acid with tetramethylammonium hydroxide. The crystal structure of the complex anion is shown in Figure 21.^31^

The [Fe^II^_4_L_6_]^4–^ complex (L = **22**) shows a regular tetrahedral geometry. The edge of the tetrahedron (Fe^II^···Fe^II^ distance) is 12.9 Å, from which a volume of 251 Å^3^ can be calculated. Such a value corresponds very roughly to the volume of the cavity. In Figure 23a, the 12 sulfonate groups covalently linked to the diphenyl spacers in 2,2′- positions are not shown for clarity. In any case, they point outward, which accounts for the solubility of the complex salt in water. However, in accordance with the Aristotelian principle (Natura abhorret vacuum), the tetrahedral cavity is not void but contains a molecule of acetone, which has been probably incorporated during the recrystallization process (diffusion of acetone on an aqueous solution of the salt).

**Figure 22:**
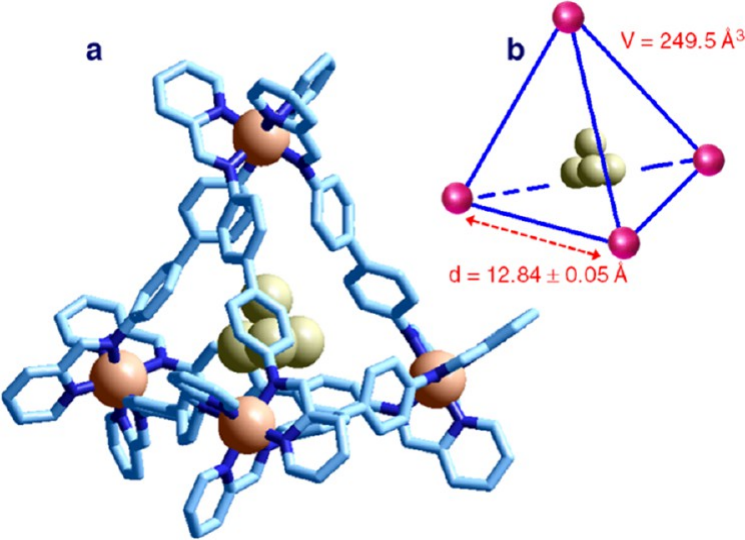
(a) Crystal structure of the salt (Me_4_N)_4_[Fe^II^_4_L_6_]·P_4_ hydrate (L = **22**).^32^ Covalently linked sulfonate groups, hydrogen atoms, tetramethylammonium ions, and solvating water molecules are omitted for clarity. A tetraphosphorus molecule is well included in the tetrahedral receptor. (b) Sketch of the tetrahedron whose vertices are occupied by Fe^II^ ions. Adapted from ref (32). Copyright 2009 American Association for the Advancement of Science.

The [Fe^II^_4_L_6_]^4–^ complex can include other molecules of appropriate size, e.g., P_4_, white phosphorus, which has a tetrahedral shape.^32^ White phosphorus is a waxy solid, soluble in apolar solvents (e.g., benzene) and insoluble in water. The sterically constrained arrangement precludes a full overlap of σ orbitals, which accounts for the formation of weak P–P bonds and for the extreme reactivity of P_4_. White phosphorus is violently pyrophoric in air with formation of P_4_O_10_ and is kept under water to avoid any contact with oxygen. When an aqueous solution of the [Fe^II^_4_L_6_]^4–^ complex is left in contact with white phosphorus, P_4_ is incorporated into the tetrahedral receptor. The crystal structure of the inclusion complex, isolated as a tetramethylammonium salt, is shown in Figure 22.^32^

The P_4_ molecule is well included in the cavity and is stabilized by the van der Waals interactions between phosphorus atoms and the aromatic rings decorating the interior of the cavity. When trapped in the cage, both in aqueous solution and in the solid phase, it is insensitive to dioxygen and remains indefinitely unchanged in air. The lack of reactivity of P_4_ does not derive from a mechanical protection exerted by the cage but by the circumstance that the reaction of O_2_ with P_4_ would necessarily generate a preliminary P=O fragment too large for the cavity. It is intriguing that the tetrahedral [Fe^II^_4_L_6_]^4–^ complex is the receptor of choice of smaller tetrahedral molecule P_4_, which suggests the existence of a principle of geometrical correspondence (like includes like). However, if an aqueous layer containing the {[Fe^II^_4_L_6_]·P_4_}^4–^ inclusion complex is equilibrated (vigorously shaken) with a benzene layer, P_4_ moves to the organic layer and is replaced in the cavity by a benzene molecule. Benzene has a shape quite different from a tetrahedron, but it can establish quite strong π–π interactions with the aromatic moieties coating the cavity’s walls, distinctly stronger than the vdW interactions established by P_4_.

## 7 An Icon of Human Image: The Double Helix

**Figure 23:**
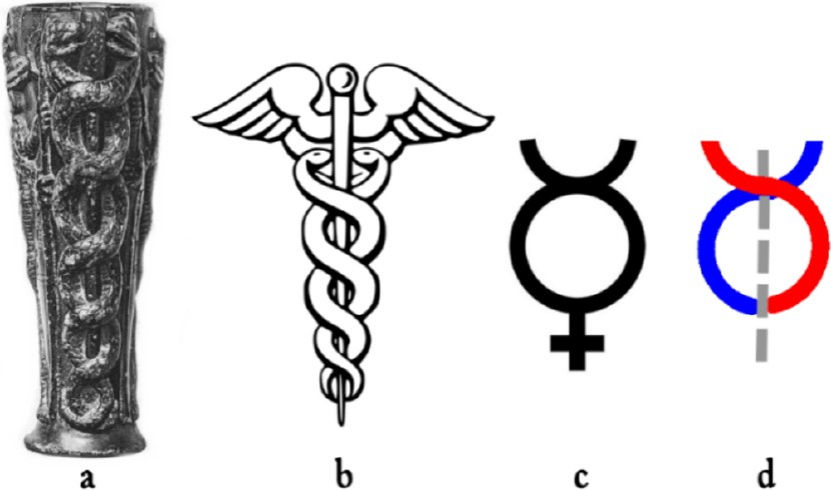
(a) “Libation vase of Gudea” dedicated to the Sumerian deity Ningišzida, the god of nature and fertility (the two snakes, male and female), coiling around an axial rod as a double helix, depict the god himself). Source: http://1886.u-bordeaux-montaigne.fr/items/viewer/76267#page/n1/mode/1up. (b) Caduceus, the short wand of Hermes (Mercury). (c) Alchemic symbol of mercury (metal), a stylized caduceus. (d) Two stylized snakes of a caduceus entwined in a double helix. Adapted from de Sarzec and Heuzey (1884). Copyright 1884 E. Leroux.

Objects arranged in a double-strand helical shape have attracted and intrigued human beings for a long time.^33^ The first known example refers to a green steatite libation vase exhibited in the Louvre featuring the Sumerian deity Ningišzida (see Figure 23a).

Gudea, the ruler of the city and state of Lagash in Southern Mesopotamia during the period 2144–2124 BC, dedicated this vase to Ningišzida, the god of fertility, represented as a pair of snakes wound around a wooden wand or a scepter. The two snakes (male and female) face each other with open mouths at the top of the staff, while at its base their tails interlace, a clear allusion to the reproductive intercourse. The theme of a staff with two snakes intertwined around it was later adopted by Greek mythology. In fact, the caduceus, a short wand entwined by two serpents with surmounting wings, is typically carried out by god Hermes (later the Latin god Mercury, shown in Figure 23b). It is believed that Hermes was an Oriental deity of Babylonian extraction, later accepted with a subsidiary role in the Olympian Pantheon. Mercury played other two significant roles in human culture: (i) as one of the seven planets of the geocentric system and (ii) as one of the seven metals of Alchemy. A stylized drawing of the caduceus (Figure 23c) represented the symbol of mercury both in astronomy and alchemy. Figure 23d highlights the intertwining of the two snakes in a double helix mode in the symbol.

**Figure 24:**
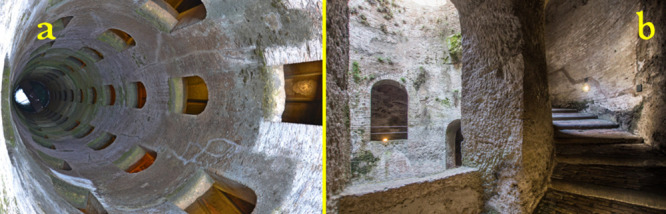
Well of Saint Patrick, Orvieto, Italy, built by the Florentine architect Antonio da Sangallo the Younger (1527–1537), under the stimulus by Pope Clemente VII, to serve as a water supply in the case of siege. (a) Top view. (b) View of the stairs of one helix [images courtesy of Bellaumbria.net].

The double helix has also represented an ambitious task in architecture: a spectacular example is provided by Saint Patrick’s well in Orvieto, Italy, designed to obtain water from the depths of the bluff where the city of Orvieto sits (Figure 24). It was constructed by Antonio da Sangallo the Younger, the most visionary architect-engineer in Italy at that time, during the period 1527–1537. The well is 53 m deep and 14 m wide and is equipped with a pair of wide spiral staircases each made of 298 stairs, lit by 72 internal windows, which forms a double helix, so that mules laden with water jars could descend on one ramp and come back up the other, without colliding.

In 1953, double helices entered astonishingly the world of science with the disclosure by Crick and Watson of the structure of DNA, a discovery that has dramatically changed the way mankind thinks about life sciences.^34^ In chemistry, the double helix has become since then a recurring motif of inspiration for molecular design and syntheses. In 1987, Lehn et al. reported the first example of inorganic double helices, i.e., a series of polynuclear metal complexes (helicates), in which two linear multidentate ligands are coiled around two or more metals forming a double helix.^35^ Both DNA and helicates are held together by labile non-covalent interactions (hydrogen bonding and metal–ligand, respectively), a feature that allows the fast and reversible molecular assembling to give an elaborate structure, through a repetitive trial-and-error mechanism. The double helical structure of helicate complexes results from the fine balance between (i) the geometrical preferences of the metal center for coordination and (ii) the steric constraints imposed by the linear ligand, which may contrast the formation of a mononuclear complex. Mononuclear tetrahedral complexes already possess a helical twist and are good candidates for the formation of helicates. In fact, the first double-stranded helicates were obtained with d^10^ metal ions (Cu^I^ and Ag^I^), which have a strong preference for a tetrahedral coordination geometry.

**Figure 25:**
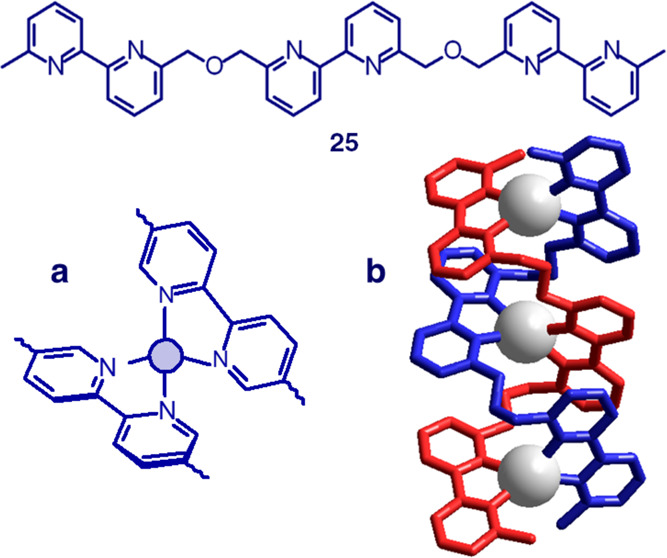
Tris-bidentate ligand containing three 2,2′-bipyridine subunits (helicands), **25**. (a) Metal ion that prefers tetrahedral coordination (d^10^ electronic configuration, e.g., Ag^I^), thus possessing a helical twist. (b) Crystal structure of the double-stranded helicate complex [Ag^I^_3_L_2_]^3+^ (L = **25**).^36^ White spheres represent Ag^I^ ions. The two strands have different colors to evidence the double-helix arrangement. Triflate counterions have been omitted for clarity.

Polypyridine **25** contains three 2,2′-bipyridine subunits (N∩N), each one capable to act as a bidentate ligand. On adding a CH_2_Cl_2_ solution of **25** to an MeCN solution of AgCF_3_SO_3_, a white precipitate of [Ag^I^_3_L_2_](CF_3_SO_3_)_3_ forms. In particular, 3 Ag^I^ ions and 2 molecules of **25** self-assemble to give trinuclear complexes in which the two polypyridine strands are intertwined around the metal ions in a double helix mode. The driving force of the process is the energy associated to the formation of a tetrahedral [Ag^I^(N∩N)_2_]^+^ complex (Figure 25a). The −CH_2_–O–CH_2_– bridge linking 2,2′-bipyridine units of **25** is too short to permit tetrahedral binding of an ion by two N∩N units of the same ligand molecule, and it is flexible enough to allow strain-free coordination in a dimeric fashion. As a consequence, the double-stranded helicate complex [Ag^I^_3_L_2_]^3+^ forms, whose crystal structure is shown in Figure 25b.^36^ On replacing AgCF_3_SO_3_ with [Cu^I^(MeCN)_4_]ClO_4_, under the same conditions, a red-orange salt precipitates of formula [Cu^I^_3_L_2_](ClO_4_)_3_, which contains a double-stranded helicate complex with a structure similar to that shown in Figure 25b.

Not unexpectedly, Cu^I^ can address Schiff base condensation of primary amines and carbonyl derivatives toward the formation of multinuclear double-stranded helicates, provided that (i) the helicand possesses sp^2^ nitrogen atoms (imines and pyridines), to favor back donation from a filled dπ orbital of the metal to an empty π* molecular orbital of the ligand, and (ii) reacting fragments are equipped with bulky substituents disfavoring the formation of mononuclear complexes.

**Figure 26:**
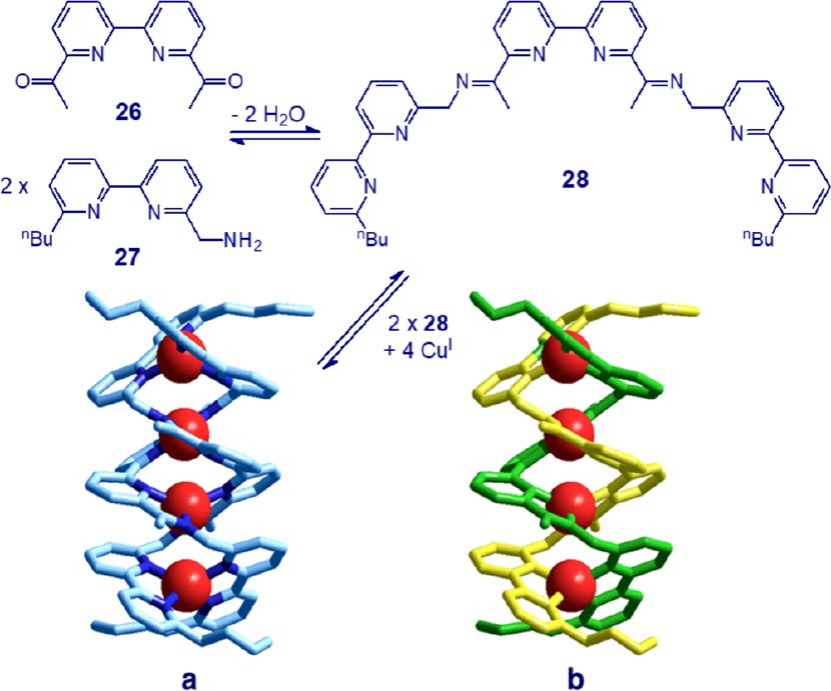
Schiff base metal template synthesis of a tetracopper(I) double-stranded helicate. (a) Crystal structure of the complex salt [Cu^I^_4_L_2_](Cu^I^_2_I_4_)_2_, L = **25**(37) (hydrogen atoms and counteranions have been omitted for clarity). (b) Same structure as a but with one strand yellow and the other green.

A good example is illustrated in Figure 26. Schiff base condensation of 1 mol of diketone **26** with 2 mol of amine **27** gives imine derivative **28**, which contains four bidentate subunits N∩N and may act as a helicand. Then, 2 mol of **28** and 4 mol of Cu^I^ assemble to give the double-stranded tetranuclear helicate complex [Cu^I^_4_L_2_]^4+^ (L = **28**), whose crystal structure is shown in Figure 26a. The four Cu^I^ ions are positioned along the axis of the helices, each one profiting from a flattened tetrahedral coordination. The four bulky *n*-butyl substituents at the end of the strands as well as the four ketamine groups prevent the formation of mononuclear species and favor the double helical arrangement.^37^

A helix possesses its own chirality depending whether it is right-handed (*P*) or left-handed (*M*). Any double helicate is a racemic mixture of the two enantiomers *P*,*P* and *M*,*M*, which are both present in the elementary cell. For instance, both the structures reported in Figure 25b, [Ag^I^_3_L_2_]^3+^, and in Figure 26, [Cu^I^_4_L_2_]^4+^, refer to *P*,*P* enantiomers.

**Figure 27:**
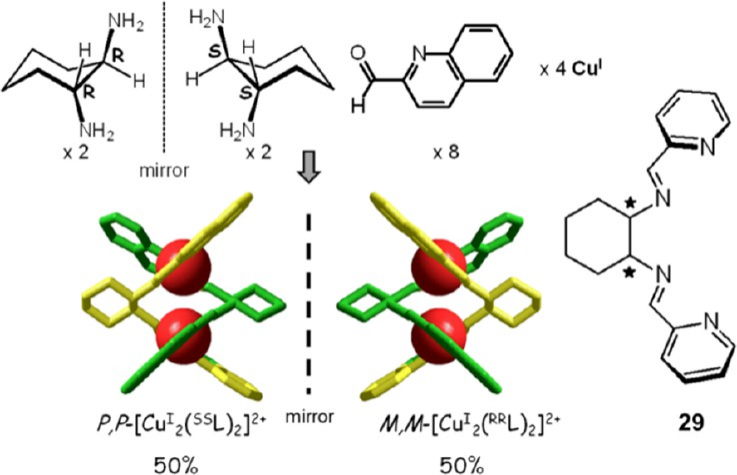
Template synthesis of the double-stranded helicate complex [Cu^I^_2_L_2_]^2+^ (L = **29**). On reaction of rac-1,2-cyclohexanediamine with isoquinolyl aldehyde, a racemic mixture of the complex was obtained consisting of the two enantiomers *M*,*M*-[Cu^I^_2_(*^RR^*L)_2_]^2+^ and *P*,*P*-[Cu^I^_2_(*^SS^*L)_2_]^2+^ in a 1:1 ratio.^38^

An intriguing complication occurs when one of the reagents of the Schiff base condensation possesses its own chirality. This is the case of the reaction of *trans*-1,2-cyclohexanediamine (racemic mixture) with 2-pyridine-carbaldehyde and its derivatives in the presence of the Cu^I^ template, illustrated in Figure 27.^38^

As the helicand **29** (= L) is constituted by two enantiomers, ^*R*,*R*^L and ^*S*,*S*^L, a further element of complexity is introduced: the matching of the chiral properties of one strand with the other when the dicopper(I) helicate complex forms. In particular, on reaction of equimolar amounts of *^rac^*L with a Cu^I^ salt, one would expect, on a pure statistical basis, the formation of a mixture of products made with 50% “scrambled” double helicate [Cu^I^_2_(*^RR^*L)(*^SS^*L]^2+^, with 25% [Cu^I^_2_(*^RR^*L)_2_]^2+^, and with 25% [Cu^I^_2_(*^SS^*L)_2_]^2+^. However, the formation of a racemic mixture of homochiral dinuclear species was observed: 50% *M*,*M*-[Cu^I^_2_(*^RR^*L)_2_]^2+^ (i.e., two intertwined helices, both with *M* handedness) and 50% *P*,*P*-[Cu^I^_2_(*^SS^*L)_2_]^2+^ (i.e., two intertwined helices, both with *P* handedness) in the unit cell. The structures of the two enantiomers are shown in Figure 27.

Thus, in the formation of the double helicate complex, strands of the same chirality seek each other, thus obeying the principle of homochiral recognition.^38,39^ The self-recognition process is described by equilibrium (eq 1)
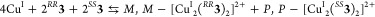
1

The occurrence of homochiral recognition has been ascribed to the fact that two rigid units of the same chirality combine to give a compact structure, whereas two heterochiral units give a less compact structure.^39^

## 8 Borromean Rings

**Figure 28:**
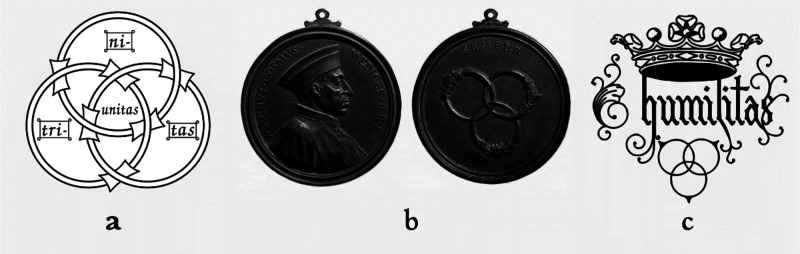
(a) Borromean rings as a symbol of the Christian Trinity, based on an illustration in a 13th-century French manuscript found at Chartres. Source: http://1886.u-bordeaux-montaigne.fr/items/viewer/76267#page/n4/mode/1up. (b) Bronze medal of unknown origin, featuring on one side Cosimo de’ Medici the Elder and on the other side the three interlocked rings, at that time the family crest. (c) Symbol of Cardinal St. Charles Borromeo (1538–1584), a prominent member of the Borromeo family (image courtesy of Seminario Arcivescovile di Milano).

Borromean Rings (BRs) are a topological object constituted by three circles: all together are bound and inseparable, but taken two by two, they are not. This basically means that if one were to cut or take away one ring, then the other two would fall apart. As such, BRs, since the early times, pictorially represented “strength in unity” and have been chosen as a symbol by several cultures and religions. As an example (see Figure 28a), in the Catholic religion, BRs represent Holy Trinity (one God in three Divine persons).

**Figure 29:**
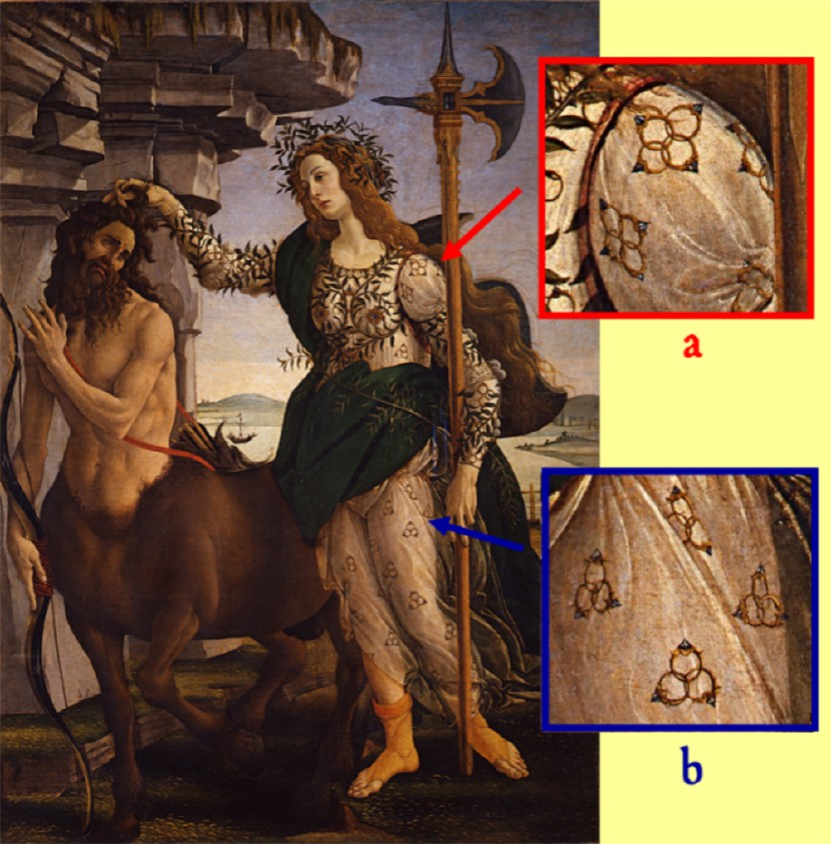
Sandro Botticelli’s (1445–1510) *Pallas and the Centaur* (ca. 1482), tempera on canvas, 207 × 148 cm, in the Uffizi Gallery, Florence. (a) Detail showing four intertwined rings. (b) Detail showing Borromean rings.

The Medici in Florence adopted the three intertwined rings as family coat of arms as shown for instance by the bronze medal featuring on one side Cosimo the Elder (1389–1464), the first member of the Medici family that de facto ruled Florence and on the other side the three rings (see Figure 29b). However, the most clear and appealing demonstration of the connection between BRs and Medici is provided by the painting by Sandro Botticelli shown in Figure 29.

The painting illustrates a subject of ancient Greek mythology: Pallas, the goddess of wisdom, clutches the hair of a centaur, and he seems submissive to her. Centaurs were typically associated with uncontrolled passion and sensuality. Thus, a first level allegory of the painting refers to the primacy of reason over passion. However, there exists a more elaborate meaning related to the political events involving the Florentine Republic and the Medici family at the end of the 15th century.

In 1478, Pope Sixtus IV formed a military alliance with King Ferdinand I of Naples, their armies invaded Tuscany, and in November 1479, they occupied Colle Val d’Elsa, a fortified town 80 km from Florence. The Florentine Republic was in serious danger, and its ruler Lorenzo de’ Medici, the Magnificent, traveled by sea to Naples to have direct talks with the King of Naples. Lorenzo was received at the court with full honors and remained in Naples as a respected guest for three months. Lorenzo impressed Ferdinand with his culture and savoir faire and ultimately convinced him to withdraw troops from Tuscany, thus ending the war. This diplomatic success highly increased in Italy and abroad the reputation of Lorenzo, who pursued a policy of maintaining peace, balancing power between Italian states, and keeping major European states such as France and the Holy Roman Empire out of Italy. Later, Medici left the three interlaced ring and took as a family crest the shield with six balls (in origin bezants, the gold coins used in the Byzantine Empire, to emphasize the main family business: banking).

Botticelli’s painting allegorically illustrates the diplomatic success by Lorenzo: the Florentine Republic, personified by Pallas, with the force of reason but also with the threat of weapons (represented by the halberd), dominates and tames the centaur, which represents the Kingdom of Naples. It is allusive of Naples the gulf in the background. The role of Medici is subtly and elegantly suggested by Pallas’ clothing, which is decorated by a variety of Borromean rings (Figure 29, inset b). The decoration was probably suggested to the painter by Lorenzo himself or by a complacent member of the Neoplatonic Circle, led by the philosopher Marsilio Ficino and the poet Agnolo Poliziano. Sandro Botticelli respectfully accepted the suggestion, but as a renowned kidder, he also put on the clothing groups of four intertwined twins, which are neither insignia nor a topological figure (Figure 29, inset b). Lorenzo and his followers took the hint but did not object.

At this stage, one could ask why we currently speak of Borromean rings and not of Medicean rings. The Borromeo family was running during the 15th century an inn in San Miniato al Tedesco, a village between Florence and Pisa, positioned along the Via Francigena, the pilgrim route running from France to Rome. Thus, the inn offered accommodation to the numerous pilgrims going to Rome (a pilgrim to Rome was called Romeo) from all of Europe. The inn was therefore entitled to “Bon Romeo” (the good pilgrim to Rome), from which the hoteliers took their surname, Borromeo. Religious tourism at that time was a big business. The Borromeo family made money enough to open a bank in Florence. They had success and opened branches in towns of Northern Italy, including Milan. Here, they found a prestigious customer, the Dukes of Lombardy, Visconti and later Sforza. These families were continuously involved in wars against the neighboring states, and in the years, they borrowed from the Borromeo bank an enormous amount of money. To service the debt, Francesco I Sforza gave to the Borromeos lands and castles in Western Lombardy, around the Lago Maggiore, and granted them the count title. At this stage, the Borromeo family was looking for a coat of arms and chose the three rings, which they had seen in Florence and which had been dismissed by Medici. Figure 28c shows the emblem of one of the most important members of the family, Cardinal St. Carlo Borromeo (1538–1584), which is still the symbol of active institutions founded by the cardinal: the Almo Collegio Borromeo in Pavia (1561) and the Archiepiscopal Seminary of Milan (1564). The word “humilitas” in the crest is taken by St. Augustine’s Latin phrase “Humilitas occidit superbiam” (humility kills pride), which refers to the victorious fight of David against the giant Goliath.

**Figure 30:**
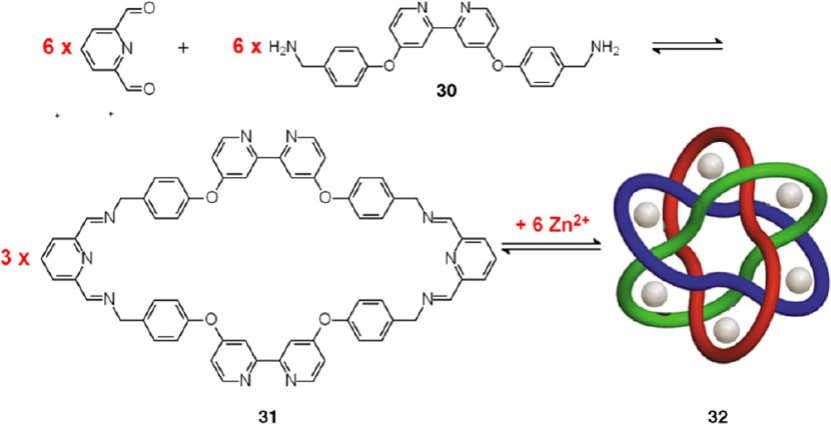
Stoddart’s template synthesis of Borromean rings.^40^ The one-pot synthesis was carried out in refluxing MeOH (3 h) in the presence of Zn(CF_3_COO)_2_. The hexametallic complex (**32**) is depicted in an orthogonal representation.

On the chemical side, BRs represented an ambitious challenge for many researchers. Classical organic synthetic procedures, made by a sequence of irreversible and kinetically controlled steps, did not produce any result. In the first years of the third millennium, Fraser Stoddart, a Scottish chemist, at that time a professor at UCLA, who in 2016 would have shared the Nobel Prize in Chemistry with Jean-Pierre Sauvage and Ben Feringa for molecular machines, adopted a metal template approach based on Schiff base condensation. The one-pot process was successful and is illustrated in Figure 30.^40^

The synthesis involves 6 mol of diformylpyridine, 6 mol of a primary diamine (**30**) containing 2,2′-bipyridine (bpy) subunits, and 6 mol of Zn^2+^ (dissolved as trifluoroacetate). Zn^II^ was chosen for its affinity toward five-coordination and was expected to bind the bidentate subunit bpy (N∩N) and the tridentate subunit (N^N^N), resulting from the condensation of diformylpyridine with the primary amine groups of two distinct molecules of **30**. (2 + 2) Schiff base condensation of diformylpyridine and **30** leads to the formation of three macrocycles (**31**). The three macrocycles, in order to ensure the formation of six [Zn(N∩N)(N^N^N)]^2+^ complex subunits, interlock themselves according to the orthogonal representation of the Borromean rings. The [Zn^II^_6_L_3_]^12+^ hexanuclear complex forms under a thermodynamic control, and its stability results from the enthalpic contributions from the formation of 30 Zn^II^–N bonds and, to a lesser extent, from the establishing of π–π interactions between aromatic rings. The reversibility of the C=N bonds and the trial-and-error mechanism allowed the achievement, in 3 h in refluxing MeOH, of a so complex and sophisticated structure. The key move of the successful game was to make the bidentate subunits N∩N point outward the macrocycle and the tridentate units N^N^N inward.

**Figure 31:**
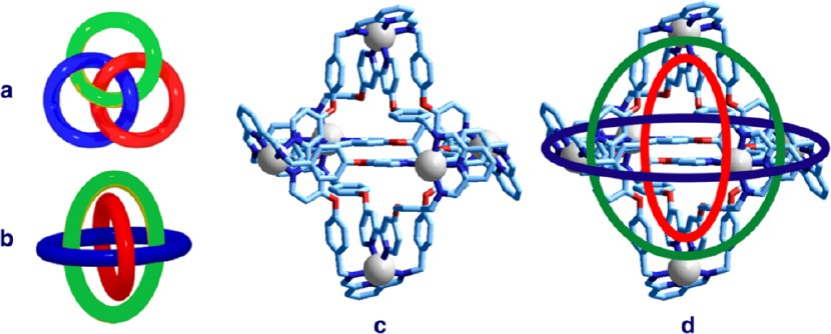
Crystal structure of the borromeate complex salt [Zn^II^_6_L_3_](CF_3_COO)_6_ (L = **31**).^40^ (a) Classical flat representation of Borromean rings. (b) Orthogonal representation (structures **a** and **b** courtesy of Mathcurve, https://mathcurve.com. (c) Crystal structure of the borromeate complex (hydrogen atoms and trifluoroacetate counterions have been omitted for clarity). (d) Same structure on which the orthogonal graph has been superimposed.

**Figure 32:**
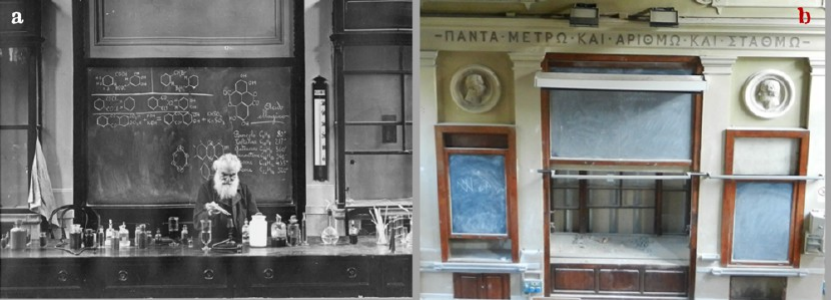
(a) Ugo Schiff delivering his last lecture (*Lectio Magistralis*) on Saturday April 24, 1915, in the amphitheater of the Institute of Chemistry at the University of Florence. (b) Front wall of the amphitheater today on refurbishment (courtesy of the Università di Firenze) (over the blackboard, there are the portraits in relief of Jakob Berzelius (1779–1848), left, and of Humphry Davy (1778–1829), right, and the Ancient Greek inscription ΠANTA METPΩ KAI APIΘMΩ KAI ΣTAΘMΩ (all things by measure and number and weight)).

Figure 31c shows the crystal and molecular structure of the [Zn^II^_6_L_3_](CF_3_COO)_12_ (L = **31**) complex salt.^42^ Taking inspiration from the nomenclature introduced by Lehn (cryptand/cryptate),^18^ the system of the three ligating macrocycles (**31**), is called borromeand (a ligand capable to give metal complexes in the form of Borromean rings) and the corresponding complex [Zn^II^_6_L_3_]^12+^ borromeate. It has to be noted that the complex appears in the orthogonal structure (Figure 31b) and not in the more familiar topologically equivalent planar structure in Figure 32a. In Figure 31d, the orthogonal graph has been superimposed on the crystal structure of the borromeate complex.

Noteworthy, despite the complexity of the mechanism, the zinc(II) borromeate salt can be obtained in a multigram scale in an undergraduate organic lab (average yield of 86%).^41^ This conclusively demonstrates the efficiency of Schiff base condensations when addressed by metal ions of known geometrical preferences and defines an emerging area in which synthetic organic, inorganic, and coordination chemistry, supramolecular chemistry, and dynamic covalent chemistry merge and cooperate.^42^

## 9 Professor Ugo Schiff: His Classroom and His Students

In 1864, Carlo Matteucci, the Minister of Public Education of the newly founded Italian State, called Hugo Schiff, a senior assistant at the University of Pisa, to cover the Chair of Chemistry at the Royal Institute for Practical and Advanced Studies in Florence, something similar to a doctorate school. At that time, there was no university in Florence. Indeed, a university had been founded in Florence as a studium in 1231, but it had been closed in 1475, transferred and merged with the University of Pisa by Lorenzo the Magnificent, a surprising resolution by a unique patron of the arts, literature, and philosophy, firmly determined to make Florence a leading center of culture in Italy and in Europe. However, Lorenzo probably had realized that universities and their students could represent a source of revolutionary ideas and a menace to the established power, i.e., something to keep at a reasonable distance (68 km in this particular case), a not odd choice at the time, if one considers that Milan had its university in Pavia (31 km), Venice in Padua (35 km), and London in Oxford (83 km) and Cambridge (80 km). Schiff, the first professor of Chemistry at the Royal Institute, found the laboratories of chemistry, hosted by the Royal Museum of Physics and Natural History, small and inadequate. He began a long fight to convince the minister to transfer Chemistry in a suitable place, which was finally founded in a former religious institute behind the Basilica of the Most Holy Annunciation, downtown. Schiff participated actively with suggestions and blueprints drawn by himself to the renovation of the old building and to its adjustment to fulfill chemical requirements (1882–1885). In particular, he personally designed the main amphitheater in which he used to have classes, inspired by the chemical amphitheater of the University of Göttingen, his alma mater. The chemical amphitheater in Florence has been eternalized by a famous photograph showing Schiff that delivers his last lecture (*Lectio Magistralis*), displayed in Figure 32a.

Figure 32b shows an inscription in ancient Greek set over the blackboard: ΠANTA METPΩ KAI APIΘMΩ KAI ΣTAΘMΩ—[You, My God, have ordered] all things by measure and number and weight. The sentence, an invocation by Solomon to God, taken from the Book of Wisdom, Chapter 11, seems to describe the divine order of the physical world and to suggest the scientific keys for studying and interpreting nature. However, on reading the complete paragraph in the Book of Wisdom, the meaning appears totally different and unrelated to science: “Even without these [the Plagues of Egypt], they [the Egyptians] could have been killed at a single breathe, pursued by justice and winnowed by Your mighty spirit. But You, My God, ordered all things by measure and number and weight.” Thus, Solomon praises the clemency of God in softly punishing the Egyptians, guilty of persecuting the Israelites. Ugo Schiff, son of a Jewish family, very probably knew the Book of Wisdom (even if this book is not accepted in the Jewish Bible), mastered ancient Greek (as well as Latin and Hebrew, plus German, Italian, and French), and was aware of the sense of Solomon’s invocation. However, he was intrigued by the “scientific” misinterpretation of the sentence and wanted it to perpetually admonish students attending classes in the amphitheater (including the writer of these notes). Significantly, the Latin version of the above sentence (Omnia in mensura et numero et pondere) is present in the Aula Magna of the Department of Chemistry at the University of Bologna. The Aula was constructed following the will of Giacomo Ciamician (1857–1922). Ciamician was a younger colleague and a friend of Ugo Schiff and was probably inspired by a visit to the chemical amphitheater in Florence.

Schiff taught classes until 1915, the year of his death, aged 81. The age limit for University professors at the time was 75, a restriction from which he was exempted for his special scientific merit. He delivered his last lecture (lectio magistralis) on April 24, 1915, the closest Saturday to his 81st birthday (April 26). Schiff chose Saturday, at the time a half working day, for not interfering with the teaching schedule of students and colleagues (see the photograph in Figure 32a).

**Figure 33:**
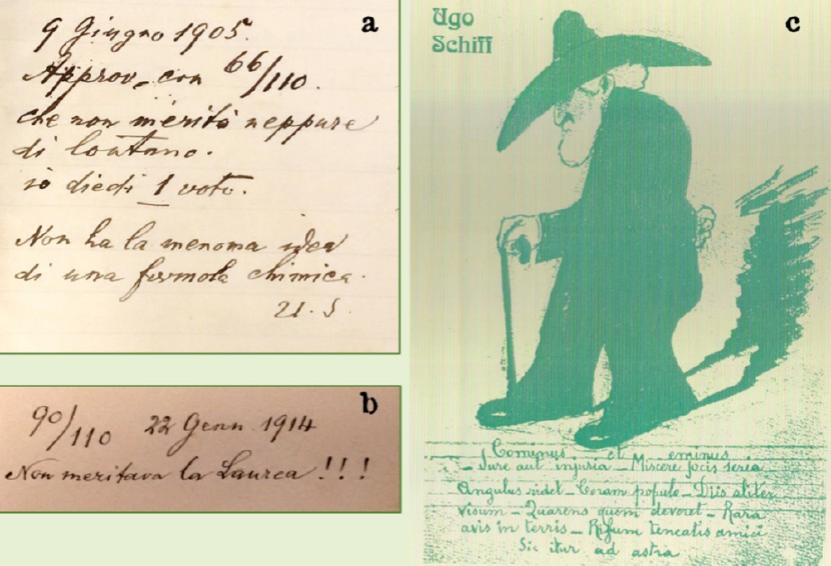
(a) Pungent handwritten note added by Schiff in the registry of graduate exams at the Royal Institute in Florence, graduation session of June 9, 1905 (TS SCHIFF 026). (b) Note of January 22, 1914 (TS SCHIFF 043). (c) Caricature of Ugo Schiff with Latin sentences that he used to tell students during classes and in the lab [courtesy of the Università di Firenze, Biblioteca di Scienze, sede Polo scientifico, and Dipartimento di Chimica Ugo Schiff].

Schiff was an appreciated and passionate teacher, but he was also a demanding examiner. Figure 33a reports a comment written by Schiff himself on the registry of graduate exams. The candidate was less than brilliant and got the minimum mark for passing exams and obtaining the degree (“laurea”): 66/110. In fact, the jury was typically constituted by 11 professors, and each member was supposed to assign a mark from 1 (very low quality) to 10 (very high quality). Schiff added this caustic note: “approved with 66/110, which [the candidate] did not deserve even remotely. I gave 1. [The candidate] has absolutely no idea of a chemical formula”. The note was signed with the initials U. S., for Ugo Schiff: the German first name Hugo had been italianized to Ugo. In another graduation session (Figure 33b), he commented, “90/110, [the candidate] did not deserve the degree!!!”.

However, students not only highly esteemed but loved their grouchy professor. This is confirmed by a cordial caricature of the old professor drawn by a student (Figure 33c). Below the sketch, there are some Latin quotations that Schiff used to tell students during classes or in the laboratory. Some examples: “Rara avis in terris, [nigroque simillima cygno]” (A rare bird upon the earth, [and exceedingly like a black swan], Juvenal, *Satires*, VI, 165) was probably said to praise the only student in the classroom able to answer a question by the professor; “Dis aliter visum” (it seemed otherwise to the gods: i.e., fate had different plans, Virgil, *Aeneid*, II, 428) was to comfort a student for the failure of an experiment; “Sic itur ad astra” (thus one goes to the stars, Virgil, *Aeneid*, IX, 641) was to applaud a student for a successful achievement in the lab.

## 10 Epilogue

The name Schiff will last in the language of chemistry as long as this discipline will be studied and practiced. “Schiff base” is one of the most frequently used expression in chemistry with an associated surname, rivaled only by “Grignard reagent”. What remains of Professor Schiff are the articles, the books, the classes that formed thousands of chemists, from his students to the students of his students, down to today students. However, Ugo Schiff gave important lessons also in the social side. The very last one was the order of his funeral service, drawn by himself and published in the local newspaper (*La Nazione*), on September 9, 1915, the day after his death: “ I order that my remains will be carried out to Trespiano [the biggest cemetery of Florence] for cremation, early in the morning, with no entourage, no speeches, no flowers, in a coffin of raw timber, with a third class hearse, more pauperum”. A final Latin quotation was more pauperum, as poor people do.
